# Omics-assisted characterization of two-component system genes from *Gossypium Raimondii* in response to salinity and molecular interaction with abscisic acid

**DOI:** 10.3389/fpls.2023.1138048

**Published:** 2023-03-31

**Authors:** Asima Rasool, Farrukh Azeem, Mahmood Ur-Rahman, Muhammad Rizwan, Muhammad Hussnain Siddique, Daniyah Habiballah Bay, Najat Binothman, Najla Amin T. Al Kashgry, Sameer H. Qari

**Affiliations:** ^1^ Department of Bioinformatics and Biotechnology, Government College University Faisalabad, Faisalabad, Pakistan; ^2^ Department of Environmental Sciences, Government College University Faisalabad, Faisalabad, Pakistan; ^3^ Department of Biology, Faculty of Applied Science, Umm Al-Qura University, Makkah, Saudi Arabia; ^4^ Department of Chemistry, College of Sciences & Arts, King Abdulaziz University, Rabigh, Saudi Arabia; ^5^ Department of Biology, College of Science, Taif University, Taif, Saudi Arabia; ^6^ Department of Biology, A1-Jumum University College, Umm A1-Qura University, Makkah, Saudi Arabia

**Keywords:** two-component system, HKS, HPS, RRs, differential expression, 3D structures, molecular-docking, ABA

## Abstract

The two-component system (TCS) genes are involved in a wide range of physiological processes in prokaryotes and eukaryotes. In plants, the TCS elements help in a variety of functions, including cell proliferation, response to abiotic and biotic stresses, leaf senescence, nutritional signaling, and division of chloroplasts. Three different kinds of proteins make up the TCS system in plants. These are known as HKs (histidine kinases), HPs (histidine phosphotransfer), and RRs (response regulators). We investigated the genome of *Gossypium raimondii* and discovered a total of 59 GrTCS candidates, which include 23 members of the HK family, 8 members of the HP family, and 28 members of the RR family. RR candidates are further classified as type-A (6 members), type-B (11 members), type-C (2 members), and pseudo-RRs (9 members). The GrTCS genes were analyzed in comparison with the TCS components of other plant species such as *Arabidopsis thaliana, Cicer arietinum, Sorghum bicolor, Glycine max*, and *Oryza sativa.* This analysis revealed both conservation and changes in their structures. We identified 5 pairs of GrTCS syntenic homologs in the *G. raimondii* genome. All 59 TCS genes in *G. raimondii* are located on all thirteen chromosomes. The GrTCS promoter regions have several *cis*-regulatory elements, which function as switches and respond to a wide variety of abiotic stresses. RNA-seq and real-time qPCR analysis showed that the majority of GrTCS genes are differentially regulated in response to salt and cold stress. 3D structures of GrTCS proteins were predicted to reveal the specific function. GrTCSs were docked with abscisic acid to assess their binding interactions. This research establishes the groundwork for future functional studies of TCS elements in *G. raimondii*, which will further focus on stress resistance and overall development.

## Introduction

The two-component system (TCS) was discovered as the crucial signaling transduction system performing key processes both in eukaryotes and prokaryotes, including plants (Mizuno, 1997; Gao and Stock, 2009). These physiological processes involve cell division, stress tolerance, chloroplast division, nutrient signaling, and leaf senescence (Hwang and Sheen, 2001; Urao et al., 2001). A number of genes assist in these processes but three types of proteins have been distinguished so far. It was first studied in bacteria. This system has two main divisions, one is the histidine kinase sensor (HK), which is membrane associated and the second is a cytoplasmic response regulator (RR) with a receiver domain called Rec domain (West and Stock, 2001). The function of HK is the direct phosphorylation of its related RR when a certain signal is received from the environment. In response, the activity of RR is regulated by this phosphorylation. The sensor HK has two domains called input and transmitter domain. The input domains collect signals and in response, the transmitter domain regulates its HK activity after the autophosphorylation of a histidine residue. Autophosphorylation is a process in which a protein kinase catalyzes the transfer of a phosphate group from ATP to a hydroxyl group on one of its amino acid residues. This process results in the activation of the kinase itself and may also enable the recruitment and activation of downstream signaling molecules (Dabravolski and Isayenkov, 2023). The receiver domain present on RR transfers the phosphate group to conserved aspartic residue. Consequently, the output domain found in RR behaves as transcription factor (Stock et al., 2000). Over time, a protein family known as histidine phosphotransfer proteins (HPs) has developed a multi-step phosphorylation process in eukaryotes. It is assumed to be a link between the histidine kinase and the response regulator for phosphoryl group transfer because of the existence of an XHQXKGSSXS motif (Schaller et al., 2008; Tiwari et al., 2021). A multifaceted TCS transduction signaling system has been characterized in eukaryotic species mainly in higher plants. In plants, this system consists of three signaling elements: histidine phosphotransfer proteins (HPs), hybrid HKs, and RRs (Urao et al., 2000; Hwang et al., 2002).

Whole genome sequencing has helped to identify TCS genes in different plant species, i.e. *A. thaliana*, *T. aestivum*, *O. sativa*, and *G. max*. The role of TCS genes in these plants has also been studied, which respond to stress. The *A. thaliana* genome contains 8 HKs, 6 HPs, and 33 RRs (Hutchison and Kieber, 2002). Ethylene- (ERS), phytochrome- (PHY), and cytokinin-receptors are all HK subgroups. Additionally, there are three HKs (CKl2/HK5, HK1s, and CKI1) that are not known to belong to any particular group in *A. thaliana*. There are numerous transmembrane domains, an input and a REC domain, as well as an H residue (conserved) that includes a transmitter domain. All these makeup HK’s general structure. Few Ethylene receptors and phytochromes lacked conserved residues and motifs such as EIN4, ETR2, and ERS2 are called divergent of HKs. This is because they are unable to execute HK activation. The ERS subgroup comprises three different domains: a HisKA, a transmembrane domain that is involved in interacting with ethylene, and a GAF that participates in the interactions of protein-protein. There are two subgroups of ERS family on the basis of similarities in the sequences of their proteins such as AETR1 and AERS1. Each of the 5 candidates of the ERS family discovered in *A. thaliana* has a C_2_H_4_ domain that facilitates the binding of ethylene. These potential candidates have been given the names ERS1, ERS2, EIN4, ETR1, and ETR2 (Ahmad et al., 2020; Chen et al., 2020b). Phytochromes are receptors that respond to light stimulation and also regulate the growth and development of plants. The phytochrome subfamily consists of five members as PHYE, PHYD, PHYA, PHYB, and PHYC. Each of the 5 receptors has an amino- and a carboxyl-terminal domain, which are the two structural regions that make up each receptor. To enhance light absorbance and photo-reversible action, a tetrapyrrole chromophore has been covalently bonded to the domain at amino terminus. Carboxyl terminal has two domains, which are referred to as PAS (2) and HisKA (1). The transmission of signals is facilitated by these domains. The three transmembrane HKs work as cytokinin receptors known as AHK2, AHK3, and AHK4. They are detected due to the presence of CHASE domain in their structure (Hutchison and Kieber, 2002; Paik and Huq, 2019).

The HP family contains a domain known as Hpt, which is essential for transferring phosphate groups from HK to the RR family. This domain must contain the conserved pattern XHQXKGSSXS (Gupta et al., 2020). The five TCS members of *A. thaliana* are known as AHP5, AHP4, AHP3, AHP1, and AHP2 (all have Hpt domain). On the other hand, AHP6’s conserved motif does not contain H residue. So, it is referred to as a pseudo HP. AHP6 was referred to as a negative regulator of the cytokinin signaling pathway because it is incapable of acting as an HP. Gene structure, conserved motifs, and domains have been used to categorize members of the RR family into 3 distinct types such as type C, type B, and type A. Type A response regulators possess REC domain and function as proteins which are responsive to cytokinin. Type B response regulators have REC domain at N-terminus and a domain at C-terminus. Cytokinin is essential for the activation of type C response regulators and they possess domain architectures identical to type-B. Pseudo response regulators, also known as PRRs are characterized by the absence of a conserved residue D. They are unable to be phosphorylated. In cytokinin signaling, when *A. thaliana* is exposed to a stressor, the cytokinin receptors make a negative response (Grefen and Harter, 2004). AHKs collect signals and are autophosphorylated in the transmitter domain at conserved histidine residues. Afterward, the conserved aspartate residues are phosphorylated with phosphoryl groups. In the end, the transmitter domain transmits the signal to the receiver domain in the RR B-type and then act as a transcription factor. In this mechanism type-B RRs activate the type-A RRs in the nucleus. In addition, type-B RRs can also bind to different *cis*-elements of the promoter genes, like stress related genes and MAPKs responding to stress (Ishida et al., 2009).

In *T. aestivum*, 62 genes of TCS have been characterized of which 7 candidates from the HK family, 10 from the HP family, and 45 from the RR family (Gahlaut et al., 2014). In *O. sativa*, 37 genes of TCS have been characterized. Both families HK and HP have the same number of members (5), however, RR family has 27 candidates (Pareek et al., 2006). TCS signaling system is the crucial pathway in the stress signaling transduction, such as salt, drought, cold, temperature, and heat (Gupta et al., 2020; West and Stock, 2001; Zwack and Rashotte, 2015; Rasul et al., 2017). In *A. thaliana*, all HKs and HPs respond either negatively or positively to salinity, cold, and drought stress. A-type RRs are involved in the osmosis-stress response by working with abscisic acid and the response may be negative or positive (Miyata et al., 1998; Zwack and Rashotte, 2015). In soybean, the dehydration-sensitive TCS genes have been characterized (Mochida et al., 2010). The role of TCS genes in tomatoes has also been studied in response to stress (He et al., 2016a).

Cotton, an economically significant crop, serves as a model for various studies and is expected to be a common source of organic fiber (Li et al., 2014). *G. raimondii* (target plant) is diploid cotton species having a D-genome (Kirungu et al., 2018; Khan et al., 2020). It is used to produce fabric, industrial moisturizers, eatable oil, and chemicals that are essential for various industries and applications (Khadi et al., 2010). It is very sensitive to drought, pathogens, and inappropriate temperature, which reduces production and fiber reliability. TCS signaling transduction system plays a key role in the stress signaling of plants (Sharif et al., 2019; Azeem et al., 2022). The present research’s goal was to uncover TCS genes in *G. raimondii* since the detailed study and identification of stress-responding TCS genes can help to combat the susceptibility and mechanism of tolerance. A comparative phylogenetic tree was generated using the protein sequences of the *G. raimondii* and five other species (*A. thaliana, G. max, C. arietinum, S. bicolor, and O. sativa*). The study employed several methods, including motif and domain analysis, *cis*-element prediction, RNA-seq data expression profiling, real-time qPCR, and molecular docking analysis to better understand the role TCS genes in *G. Raimondii* stress response.

## Material and methods

### Identification of two-component system genes in *Gossypium raimondii*

Protein sequences of already known two-component system genes in *A. thaliana* were taken from the Ensemble Plants database (https://plants.ensembl.org/index.html). These sequences were used as queries to perform the NCBI-BLASTp program against the target species (*G. raimondii*) to identify TCS genes (Mahram and Herbordt, 2010). All identified genes were analyzed by using different databases i.e. SMART (http://smart.embl.de/) (Schultz et al., 2000), CDD (https://www.ncbi.nlm.nih.gov/Structure/cdd/wrpsb.cgi) (Marchler-Bauer et al., 2015) and Pfam (https://pfam.xfam.org/) (Finn et al., 2009). The purpose of this analysis was to validate the identified sequences that must contain TCS-specific domains which are needed to perform normal TCS functions. Genomic information retrieved from NCBI for additional analysis, including protein length, chromosomal location and the number of exons and introns. Furthermore, the ExPASY ProtParam program (https://web.expasy.org/protparam/) was used to determine physiochemical characteristics such as molecular weight, isoelectric point, instability index, aliphatic index, and GRAVY (Gasteiger et al., 2005). TMHMM 2.0 (https://services.healthtech.dtu.dk/service.php?TMHMM-2.0) (Chen et al., 2003) and CELLO v.2.5 (http://cello.life.nctu.edu.tw/) (Yu et al., 2006) were used to identify transmembrane domains and subcellular localizations respectively.

### Prediction of gene structure and conserved motifs

Gene Structure Display Server (GSDS) (http://gsds.gao-lab.org/) (Hu et al., 2015) was used to display the intron-exon patterns. Genomic and coding sequences of TCS genes from the target species were used for this objective. MEME program (https://meme-suite.org/meme/tools/meme) was employed to identify the particular conserved motifs of identified sequences. All default settings were used except the number of motifs which was fixed at 20 (Bailey et al., 2006).

### MSA, phylogenetic, and putative promoter region analysis

To comprehend the ancestral relationship of GrTCS genes, the identified sequences were aligned with reference sequences using ClustalW (https://www.genome.jp/tools-bin/clustalw), a multiple sequence alignment program (Thompson et al., 2003). The phylogenetic study was carried out using the MEGA7 (Kumar et al., 2016) and IQ-TREE (http://iqtree.cibiv.univie.ac.at/) programs (Nguyen et al., 2015). The Neighbor-joining and Maximum Likelihood approaches have been utilized to generate the phylogenetic tree, which had 1000 bootstrap values. The iTOL (https://itol.embl.de/) (Letunic and Bork, 2007) web application was used to create an interactive representation of the phylogenetic tree. NCBI database was used to extract upstream genomic sequences (1,000 bp) of *G. raimondii’s* TCS genes. These sequences were input into plantCARE online program (https://bioinformatics.psb.ugent.be/webtools/plantcare/html/) to find out what possible *cis*-regulatory elements they had (Lescot et al., 2002).

### Chromosomal localization, gene duplications, and syntenic analysis

GrTCS chromosomes map of genetic relationships was created employing TBtool (advanced Circos) (Chen et al., 2022). In addition, the NCBI database was utilized to determine the chromosomal locations of all identified genes in *G. raimondii*. Duplication occurrences were found by using the offline DNAsp tool. Ka and Ks were calculated to estimate the selection pressure on the duplicated GrTCS genes. We computed the divergence time using the following equation: Time = Ks/2x (x = 6.56 109^–9^) years (Mushtaq et al., 2021).

### Differential expression analysis of TCS genes in *Gossypium raimondii*

To investigate the expression profiles of putative TCS genes in G. raimondii under salt and cold stress conditions, we used the NCBI-SRA database (https://www.ncbi.nlm.nih.gov/sra) (Leinonen et al., 2011) to download two transcriptomes for comparative analysis: PRJNA601953 (BioProject’s accession of salt stress) and PRJNA554555 (BioProject’s accession of salt and cold stress) (https://www.ncbi.nlm.nih.gov/assembly/GCF000003195.3/) was used to get the annotated genome with an extension of.gtf and.fna. The G. raimondii genome sequence indices were generated using Bowtie2, and the resulting pair-end clean reads were mapped to the G. raimondii genome(Langmead and Salzberg, 2012). Cufflinks tool was used to determine the expression levels of the annotated genes in the target genome (Ghosh and Chan, 2016). Each GrTCS’s normalized FPKM value was calculated. The heatmap for differently expressed genes was created using TBtool (Chen et al., 2020a).

### Growth conditions, plant materials, and real-time qPCR

For the confirmation of RNA-seq based expression pattern, *G. raimondii* plants were grown in a climate-controlled growth chamber (16h light & 8h dark) at 30 °C and 26°C in light and dark, respectively (Azeem et al., 2022). Plants were treated with 250mM NaCl to impose salt stress (After the expansion of the first true leaf). For cold stress treatment, plants were kept at 4 °C. Leaf samples (three biological replicates) were collected at 12h after stress. The gene-specific primers were designed by using the online tool “Oligo Calculator” (http://mcb.berkeley.edu/labs/krantz/tools/oligocalc.html) and primer specificity was verified by NCBI PrimerBLAST program (https://www.ncbi.nlm.nih.gov/tools/primer-blast/. An internal control gene (*GrActin*) was used for normalization of expression (Sun et al., 2015). SYBR Green (iTaq Universal Super Mix) and CFX96 Touch™ Real-Time PCR Detection System were used for qRT-PCR analysis using the 2^−ΔΔCT^ method (Livak and Schmittgen, 2001).

### Structure prediction of differentially expressed genes

Structure prediction is essential to reveal the specific function of identified elements. Swiss-model (homology modeling server) (https://swissmodel.expasy.org/interactive) (Schwede et al., 2003) and I-TASSER (threading approach-based server) (https://zhanggroup.org/I-TASSER/) (Yang et al., 2014) were used in order to make predictions about the 3D structures of significantly up-regulated, down-regulated, and zero expression GrTCS genes in response to salt and cold stresses. After then, these structures validate by using another online tool Saves (https://saves.mbi.ucla.edu/). These structures were further refined by utilizing the online server Galaxy refine located on Galaxy-web (https://galaxy.seoklab.org/cgi-bin/submit.cgi?type=REFINE).

### Docking of differentially expressed genes

We used Moe (Vilar et al., 2008) to find the top interacting GrTCS genes. Abscisic acid (ABA) was used as a ligand to dock against the structures of these genes. It is a hormone that aids in plant growth and development. Its structure was retrieved from PubChem online database (https://pubchem.ncbi.nlm.nih.gov/) (Kim et al., 2016).

## Results

### Identification of TCS elements in *Gossypium raimondii*

The primary goal of this research was to evaluate the TCS in *G. raimondii*. We found 59 genes associated with *G. raimondii’s* TCS using whole-genome sequencing. For this purpose, We used the BLASTp program to search for TCS genes in *G. raimondii* by using the known TCS elements of *A. thaliana* (47 members) as query sequences. All found genes were given names based on the names of *A. thaliana* TCS genes, and then they were categorized into three subfamilies: Histidine Kinase (23 members), Histidine Phosphotransfer (8 members), and Response Regulator proteins (28 members) (**Table 1**).

**Table 1:** A list of the TCS genes from various plants that have been identified.

Species	Histidine-Kinase (HK)	Histidine-Phosphotransfer (HP)	Response-Regulators (RR)	Total
*A. thaliana*	8	6	33	47
*O. sativa*	5	5	27	37
*T. aestivum*	7	10	45	62
*G. max*	36	13	49	98
*C. melo L.*	17	9	25	51
*Zea mays*	11	9	39	59
*S.bicolor*	13	5	19	37
*C. arietinum*	18	7	26	51
*Z. latifolia*	25	8	36	69
*G. raimondii* (Current research)	23	8	28	59

### HK protein family in *Gossypium raimondii*

**Figure 1:**
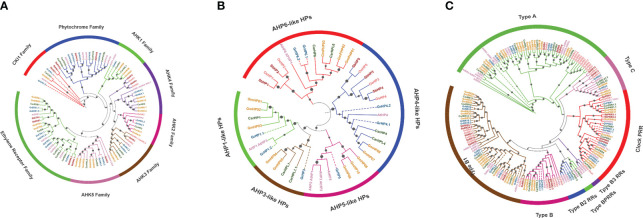
Phylogenetic evaluation of HK **(A)**, HP **(B)** and RR **(C)** proteins from *A*. *thaliana, S. bicolor, O. sativa, C. arietinum, G. raimondii and G. max*. In MEGA X, neighbor-joining methodology of 1000 bootstraps was utilized to create the tree. Particular colors are used to symbolize each species and subgroups. Candidates from GrTCS are denoted by a dashed-lines and dark blue color.

**Figure 2:**
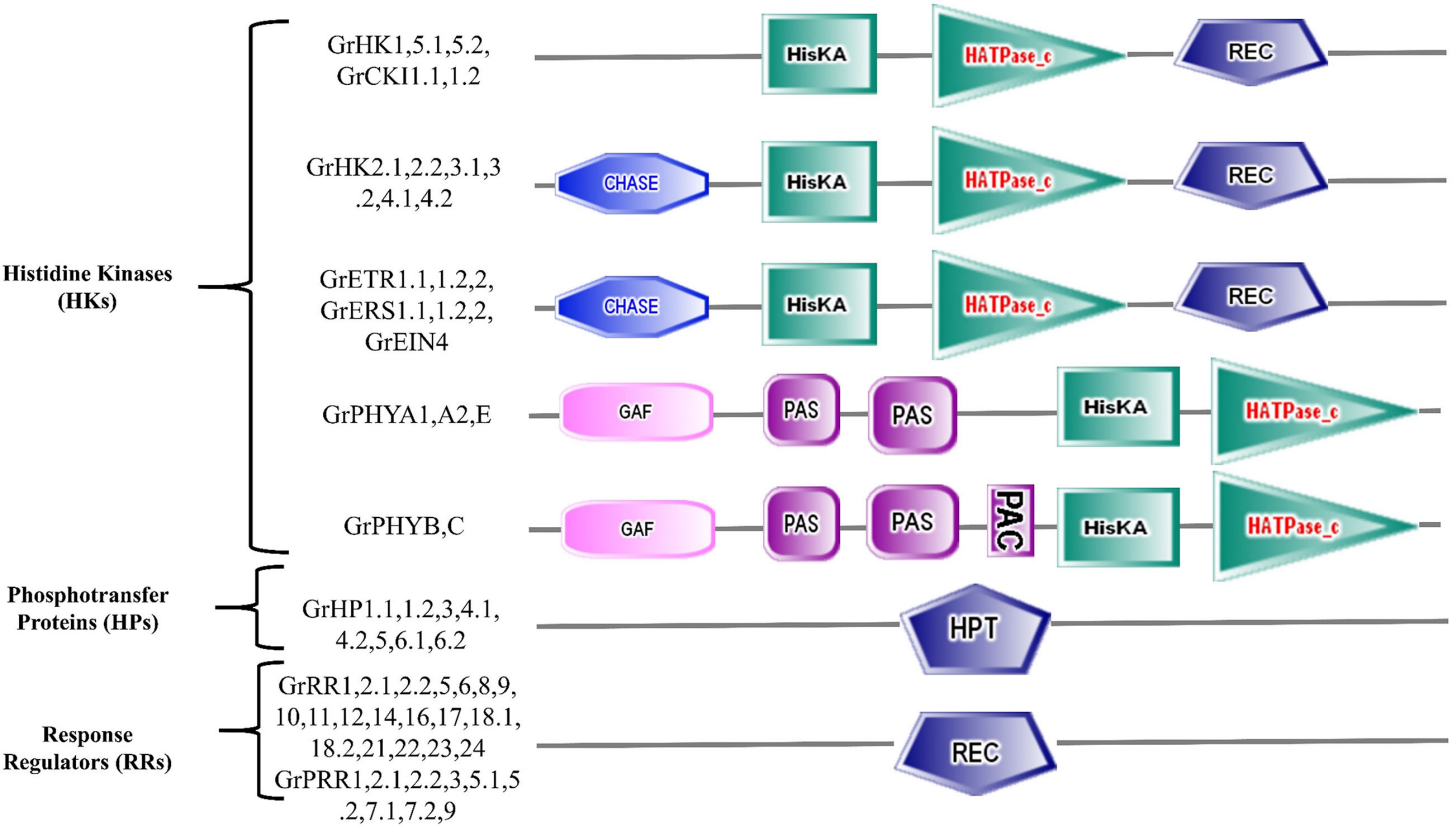
Symbolic representation of GrTCS protein domains.

*G. raimondii* has 23 members of HK family in its genome (**Table 1**). GrHKs are much more numerous than other HKs reported in non-leguminous plants: *T. aestivum* has 7 HKs, *A. thaliana* and *O. sativa* have 8 HKs, and *S. bicolor* has 13 HKs. In contrast, other leguminous plants, such as *G.max* (21), *C. arietinum* (18), and *C. melo L.* (17) contain a comparable amount of HK elements, suggesting their value in the plants (**Table 1**, **Figure 1A**). The three subgroups of the HK family are AHK, Ethylene receptor, and Phytochrome. AHK is composed of 11 members from the HK family: GrHK1, GrHK2.1, GrHK2.2, GrHK3.1, GrHK3.2, GrHK4.1, GrHK4.2, GrHK5.1, GrHK5.2, GrCKI1.1, and GrCKI1.2 that are larger than the model plant *A. thaliana* (6). They all have a HisKA domain with site of His phosphorylation, a HATPase_c domain, and a photoreceptor-like REC domain. The Cytokinin receptors GrHK2.1, GrHK2.2, GrHK3.1, GrHK3.2, GrHK4.1, and GrHK4.2 have an extra TM domain and the CHASE domain which serve as binding of Cytokinin (**Figure 2**).

Ethylene is a plant hormone that aids in aging, growth and development. Ethylene receptor elements are required for a variety of functions such as leaf abscission, propagation of root and shoot, fruit ripeness and softness. The ethylene response is mediated by 5 ethylene receptors in the model plant *A. thaliana. G. raimondii*, on the other hand, possesses seven ethylene receptor elements: GrETR1.1, GrETR1.2, GrETR2, GrERS1.1, GrERS1.2, GrERS2, and GrEIN4. *G. raimondii’s* ethylene receptors contain a HisKa domain, GAF (protein-protein interaction) domain, a HATPase_c domain (excluding GrEIN4), and a REC domain (except GrERS1.1 and GrERS1.2) (**Figure 2**).

Plants use phytochromes as photoreceptors to respond to photons and promote plant growth and development. In *A. thaliana*, candidates of phytochrome family have been identified as AtPHYA, AtPHYB, AtPHYC, AtPHYD, and AtPHYE. Phytochromes have a PHY domain at the N-terminus that implicated in the absorbing of light, a HisKa domain, two PAS domains that potentially engaged in transmission of signal, and a GAF domain. The structure of the sensor H proteins is comparable to that of soluble proteins like phytochromes. At the C-terminal, they have a HisKa domain for transmission of signal and at the N-terminal, there is a sensor domain. This family is termed as divergent HKs because it lacked the necessary 5 conserved motifs. They possess additional Ser/Thr kinase activity rather than HK activity. In *G. raimondii*, five phytochrome candidates were identified (GrPHYA1, GrPHYA2, GrPHYB, GrPHYC and GrPHYE). These members have HisKa, PHY, GAF, PAS and HATPase c domains required for light and transmission of signals, indicating that they were real photoreceptors (**Figure 2**).

### HP protein family in *Gossypium raimondii*

The Histidine phospho-transfer protein family has six members in *A. thaliana* (AHP1-AHP6). Due to the existence of H conserved residue, the first five candidates are real Histidine phospho-transfer proteins. AHP6 is referred to as pseudo HP because it lacks the H residue required for phosphate acquisition from donor protein (Bleecker, 1999). Each AHPs candidate (excluding AHP6) has a phosphorylation pattern that is conserved (XHQXKGSSXS). The N residue in AHP6 replaces the H residue in the phosphorylation motif. GrHP1.1, GrHP1.2, GrHP3, GrHP4.1, GrHP4.2, GrHP5, GrHP6.1, and GrHP6.2 were identified as HP candidates with an HPt conserved domain in *G. raimondii* (**Figure 1B**). First six members (GrHP1.1-GrHP5) have phosphorylation motif with H residue. So, they were considered real HPs. Remaining two candidates (GrHP6.1, GrHP6.2) were labelled as pseudo HPs since their H residue replaces with N residue in phosphorylation motif.

### RR protein family in *Gossypium raimondii*

The ultimate response to many environmental stimuli is regulated by response regulators (RR family). In *G. raimondii*, twenty-eight candidates of response regulator family both typical and pseudo were found. *A. thaliana* and *O. sativa* are model plants, having 33 and 22 RRs, respectively (**Table 1**) (Du et al., 2007). In the TCS signalling cascade, response regulators function as terminal components and they are switches which regulate the phosphorylation. Due to this key function, they catalysis the transfer of phosphoryl group to the conserved domain’s Asp residue. In *A. thaliana*, RRs have conserved residues such as aspartic acid (D) and lysine (K). The Rec domain contains these residues.

There are three sub-types of RR family (type-A, type-B, and type-C) on the basis of conserved domains. Type-A of response regulators possess REC domain (conserved residue D) and also contain a C-terminal extension. Type-B response regulators have two essential domains: REC domain and Myb (DNA binding) domain. Type-C response regulators exhibit a pattern comparable to type-A but lack a C-terminal extension. Some response regulators are considered as PRRs (pseudo-RRs) because they do not have D conserved residue (replaces with E residue) in REC domain and contain a CCT motif at the C-terminus. In *G. raimondii*, from 28 RRs six, eleven, two, and nine belong to type-A, type-B, type-C and PRRs respectively (**Figure 1C**).The GrRR5, GrRR6, GrRR8, GrRR9, GrRR16 and GrRR17 were discovered as type-A RR candidates in *G. raimondii*. These type-A GrRRs, like their counterparts (*A. thaliana* RRs), have a REC conserved domain (**Figure 2**). The type-B RR’s most typical candidates are nuclear proteins. They vary from type-A RRs in that they have a Myb (DNA-binding) domain. They have been shown to serve as transcriptional factors. *G. raimondii* has eleven candidates of type-B response regulators: GrRR1, GrRR2.1, GrRR2.2, GrRR10, GrRR11, GrRR12, GrRR14, GrRR18.1, GrRR18.2, GrRR21 and GrRR23. This is greater than their counterparts *O. sativa L.* (7) *Z. mays* (9) but lower than model plant *A. thaliana* (12). Myb and REC both domains are present in all type-B GrRR members excluding GrRR2.1 and GrRR23 (**Figure 2**). *G. raimondii* has two candidates for the type-C response regulator family (GrRR22 and GrRR24), which are similar to their counterpart *A. thaliana*. They contain a REC domain similar to type-A response regulators but have a short C-terminus (**Figure 2**). Phylogenetic study revealed that type-C RRs are not closely linked to type-A candidates and do not respond to cytokinin receptors.

In many plants, there is an additional subtype of RRs referred to as PRRs (diverging RRs). Model plant *A. thaliana* contains 9 PRRs with REC domain but they don’t have the DDK (conserved motif). Similarly, *G. raimondii* possesses nine PRRs named GrPRR1, GrPRR2.1, GrPRR2.2, GrPRR3, GrPRR5.1, GrPRR5.2, GrPRR7.1, GrPRR7.2, and GrPRR9. On the basis of their C-terminus extension, divergent RRs are classified into two types such as type-B PRRs contain two candidates (GrPRR2.1, 2.2) with Myb domain and clock PRRs possesses seven members: GrPRR1, GrPRR3, GrPRR5.1, GrPRR5.2, GrPRR7.1, GrPRR7.2, and GrPRR9. All clock PRRs have a CCT motif.

### Genomic and physiochemical parameters of the GrTCS elements

All genomic and physiochemical features of fifty-nine identified GrTCS elements are exhibited in **Table 2**. These genes are located at one to thirteen chromosomes. There are a large number of GrTCS genes (12) on chromosome 7. They have 2-17 and 1-16 exons and introns, respectively. Protein length of GrTCS genes varied from 134 to 1275 amino acids. Physical and chemical parameters of GrTCS proteins such as molecular weight, pI, instability index, aliphatic index, and GRAVY are ranging from 15029.42 to 142735.62, 4.67 to 9.25, 28.10 to 71.06, 63.68 to 108.69 and -0.777 to 0.185, respectively. GrTCS elements are present in the nuclear, mitochondrial, and plasma membranes (**Table 2**).

**Table 2:** Nomenclating, physicochemical characteristics, and genomic features of GrTCS proteins.

Sr#	Gene Name	Protein ID	Gene symbol	Locus Tag	TM domains	Start site	End site	CDS	Protein length	Chr#	Exons	Introns	Molecular weight (D)	pI	Instability index	Aliphatic index	GRAVY	Cell location	Str-and
1	GrHK1	XP_012469310.1	LOC105787464	B456_003G008400	2	503612	510356	4014	1231	3	13	12	136589.75	6.93	43.24-unstable	93.42	-0.190	Plasma Membrane	Reverse
2	GrHK2.1	XP_012485876.1	LOC105799728	B456_001G192400	3	32445260	32454789	5393	1275	1	16	15	142735.62	6.75	44.82-unstable	90.35	-0.217	Plasma Membrane/Nuclear	Forward
3	GrHK2.2	XP_012491050.1	LOC105803420	B456_007G166900	2	14829312	14837655	4444	1244	7	17	16	139239.16	7.24	43.25-unstable	92.36	-0.204	Nuclear/Mitochondrial/Plasma Membrane	Forward
4	GrHK3.1	XP_012479125.1	LOC105794486	B456_005G165600	3	48596083	48602783	4604	1025	5	11	10	114559.67	7.05	37.32-stable	90.61	-0.136	Plasma Membrane	Reverse
5	GrHK3.2	XP_012438330.1	LOC105764343	B456_008G165600	2	43500084	43508600	4389	1014	8	11	10	113692.26	6.89	40.31-unstable	89.87	-0.160	Plasma Membrane	Forward
6	GrHK4.1	XP_012463151.1	LOC105782746	B456_013G031500	2	2366024	2372760	4238	1003	13	11	10	111179.64	6.62	40.35-unstable	86.35	-0.172	Mitochondrial/Cytoplasmic	Reverse
7	GrHK4.2	XP_012438397.1	LOC105764395	B456_008G170200	2	44430922	44437289	3346	1006	8	12	11	111402.17	7.83	39.50-stable	87.74	-0.146	Mitochondrial	Reverse
8	GrHK5.1	XP_012444156.1	LOC105768649	B456_009G066300	0	4714872	4720202	3143	1017	9	12	11	114181.82	5.11	53.35-unstable	80.95	-0.496	Nuclear	Reverse
9	GrHK5.2	XP_012444154.1	LOC105768647	B456_009G066000	0	4694607	4701762	4444	1018	9	13	12	114633.23	5.16	51.96-unstable	80.31	-0.518	Nuclear	Reverse
10	GrCKI1.1	XP_012435523.1	LOC105762170	B456_008G139900	2	39057171	39063182	3257	1058	8	7	6	117096.87	6.37	35.77-stable	91.74	-0.152	Plasma Membrane	Reverse
11	GrCKI1.2	XP_012488043.1	LOC105801271	B456_007G305700	2	51906383	51910353	3470	1096	7	7	6	122064.41	6.25	37.02-stable	96.58	-0.148	Plasma Membrane	Forward
12	GrETR1.1	XP_012453888.1	LOC105775948	B456_002G038300	5	3054829	3059066	2912	785	2	6	5	88339.66	7.34	42.76-unstable	108.22	0.185	Plasma Membrane	Reverse
13	GrETR1.2	XP_012441097.1	LOC105766259	B456_009G358200	3	47039268	47045088	3124	740	9	8	7	82819.97	7.12	40.88-unstable	108.65	0.123	Plasma Membrane	Forward
14	GrETR2	XP_012487258.1	LOC105800596	B456_006G247800	3	49260642	49264377	3162	764	6	3	2	85132.77	7.53	40.03-unstable	101.79	0.027	Plasma Membrane	Forward
15	GrERS1.1	XP_012438019.1	LOC105764096	B456_008G143100	3	39431492	39435246	2375	637	8	6	5	71433.38	6.64	42.64-unstable	108.07	0.121	Plasma Membrane	Forward
16	GrERS1.2	XP_012490382.1	LOC105802997	B456_007G126300	3	10085905	10089617	2517	636	7	6	5	71470.35	6.48	38.77-stable	108.69	0.128	Plasma Membrane	Forward
17	GrERS2	XP_012481952.1	LOC105796708	B456_005G047900	3	4636015	4640151	3355	776	5	3	2	86345.15	6.84	44.54-unstable	105.15	0.064	Plasma Membrane	Forward
18	GrEIN4	XP_012492822.1	LOC105804671	B456_007G280100	4	47993587	47998239	3705	761	7	3	2	85229.92	6.80	37.74-stable	101.16	0.033	Plasma Membrane	Forward
19	GrPHYA1	XP_012462955.1	LOC105782636	B456_013G203900	0	51479839	51484397	3867	1122	13	5	4	124508.21	5.91	46.75-unstable	92.97	-0.113	Cytoplasmic	Forward
20	GrPHYA2	XP_012493004.1	LOC105804796	B456_007G292800	0	50235404	50241054	3873	1123	7	5	4	124756.35	5.78	45.27-unstable	94.17	-0.115	Cytoplasmic	Forward
21	GrPHYB	XP_012455449.1	LOC105776987	B456_011G200200	0	48430600	48436196	4179	1196	11	5	4	133319.94	6.07	48.01-unstable	89.28	-0.184	Cytoplasmic	Forward
22	GrPHYC	XP_012491032.1	LOC105803411	B456_007G166300	0	14686312	14691704	4178	1083	7	4	3	120618.23	5.39	52.58-unstable	91.11	-0.147	Cytoplasmic/Nuclear	Forward
23	GrPHYE	XP_012454413.1	LOC105776357	B456_011G040900	0	3037380	3041869	4014	1128	11	5	4	125901.41	5.80	44.44-unstable	92.32	-0.165	Cytoplasmic	Reverse
24	GrHP1.1	XP_012455061.1	LOC105776739	B456_011G182900	0	43533528	43535167	915	154	11	7	6	17585.15	4.80	49.41- unstable	93.64	-0.181	Cytoplasmic/Nuclear	Forward
25	GrHP1.2	XP_012487408.1	LOC105800687	B456_006G256600	0	49864408	49866269	807	154	6	6	5	17580.11	4.67	55.33- unstable	97.40	-0.076	Cytoplasmic	Forward
26	GrHP3	XP_012492998.1	LOC105804793	B456_007G291700	0	49956633	49958444	987	149	7	7	6	17364.57	4.86	48.01- unstable	90.20	-0.430	Nuclear	Reverse
27	GrHP4.1	XP_012442581.1	LOC105767560	B456_009G137400	0	10374567	10376117	904	150	9	6	5	17543.13	9.25	36.54-stable	71.60	-0.725	Nuclear	Forward
28	GrHP4.2	XP_012449005.1	LOC105772237	B456_010G104900	0	19152668	19154831	821	144	10	6	5	16776.75	5.61	57.34- unstable	73.82	-0.685	Nuclear	Reverse
29	GrHP5	XP_012490490.1	LOC105803070	B456_007G132900	0	10778947	10781363	825	153	7	6	5	17374.70	5.14	41.88- unstable	89.80	-0.263	Nuclear	Reverse
30	GrHP6.1	XP_012483242.1	LOC105797911	B456_006G101300	0	34143635	34145410	842	156	6	5	4	18083.72	6.20	52.33-unstable	105.06	-0.244	Extracellular/Nuclear	Reverse
31	GrHP6.2	XP_012449172.1	LOC105772458	B456_010G225500	0	59748642	59750621	829	156	10	5	4	18090.75	7.01	46.36-unstable	95.06	-0.256	Extracellular/Nuclear	Forward
32	GrRR1	XP_012487455.1	LOC105800716	B456_006G259700	0	50069659	50073127	2671	672	6	7	6	74145.45	6.00	49.31-unstable	75.74	-0.508	Nuclear	Reverse
33	GrRR2.1	XP_012453261.1	LOC105775279	B456_002G029300	0	2181758	2184016	650	153	2	4	3	17113.82	6.07	44.00-unstable	91.05	-0.134	Cytoplasmic/Nuclear	Forward
34	GrRR2.2	XP_012490373.1	LOC105802991	B456_007G125800	0	10040992	10043243	1884	434	7	5	4	48371.01	5.19	44.13-unstable	89.84	-0.289	Nuclear	Reverse
35	GrRR5	XP_012458513.1	LOC105779303	B456_012G123500	0	28550704	28552504	1479	236	12	5	4	26098.45	5.50	66.91-unstable	82.58	-0.528	Nuclear	Forward
36	GrRR6	XP_012486301.1	LOC105800006	B456_006G187400	0	44424672	44426990	1290	209	6	5	4	22879.05	6.97	71.06-unstable	84.83	-0.387	Nuclear	Reverse
37	GrRR8	XP_012460026.1	LOC105780318	B456_012G017600	0	2008752	2009956	1122	189	12	2	1	21282.05	5.34	49.87-unstable	82.96	-0.567	Nuclear	Reverse
38	GrRR9	XP_012461152.1	LOC105781126	B456_013G004700	0	351616	352630	852	166	13	3	2	18846.73	7.75	40.96-unstable	92.71	-0.367	Nuclear	Forward
39	GrRR10	XP_012451762.1	LOC105774016	B456_010G243900	0	61308218	61311851	2347	615	10	6	5	67140.76	6.40	36.91-stable	81.82	-0.466	Nuclear	Forward
40	GrRR11	XP_012479245.1	LOC105794555	B456_005G173300	0	50306554	50310177	2180	573	5	5	4	64343.78	5.12	42.12-unstable	81.48	-0.408	Nuclear	Reverse
41	GrRR12	XP_012444721.1	LOC105768958	B456_009G061000	0	4372520	4376660	2445	659	9	6	5	72439.72	5.65	42.27-unstable	84.02	-0.418	Nuclear	Forward
42	GrRR14	XP_012478974.1	LOC105794369	B456_005G156900	0	44464539	44470285	2896	669	5	6	5	72631.74	6.35	55.03-unstable	80.84	-0.358	Nuclear	Reverse
43	GrRR16	XP_012448024.1	LOC105771160	B456_009G294000	0	25540837	25542147	926	162	9	5	4	18091.95	5.27	38.39-stable	90.74	-0.224	Nuclear	Forward
44	GrRR17	XP_012457849.1	LOC105778654	B456_011G210000	0	50613562	50614853	943	153	11	5	4	16825.64	5.44	42.05-unstable	95.49	-0.113	Nuclear/Cytoplasmic	Forward
45	GrRR18.1	XP_012441162.1	LOC105766286	B456_001G030600	0	2866711	2870395	2991	665	1	6	5	73057.70	5.47	35.84-stable	78.00	-0.508	Nuclear	Forward
46	GrRR18.2	XP_012446887.1	LOC105770299	B456_009G054200	0	3936833	3940543	2766	697	9	6	5	76154.57	5.59	36.57-stable	70.60	-0.598	Nuclear	Forward
47	GrRR21	XP_012440210.1	LOC105765572	B456_008G280500	0	55739994	55744526	2606	782	8	10	9	83873.36	5.53	41.87-unstable	74.35	-0.500	Nuclear	Reverse
48	GrRR22	XP_012474686.1	LOC105791244	B456_004G125100	0	32611795	32613280	795	146	4	3	2	15834.17	4.87	28.10-stable	98.08	-0.042	Chloroplast	Reverse
49	GrRR23	XP_012435685.1	LOC105762426	B456_008G116600	0	35059366	35063913	1224	314	8	5	4	35313.46	6.80	42.75-unstable	98.09	-0.307	Nuclear	Forward
50	GrRR24	XP_012477997.1	LOC105793697	B456_004G223600	0	55819263	55820057	697	134	4	2	1	15029.42	5.94	48.07-unstable	91.64	-0.303	Nuclear/Cytoplasmic	Forward
51	GrPRR1	XP_012470783.1	LOC105788428	B456_003G098300	0	30484197	30489559	2740	552	3	6	5	61878.97	5.66	58.46-unstable	65.67	-0.757	Nuclear	Forward
52	GrPRR2.1	XP_012472126.1	LOC105789333	B456_003G180100	0	45178691	45183949	2168	537	3	14	13	59969.22	5.48	49.85-unstable	63.89	-0.777	Nuclear	Forward
53	GrPRR2.2	XP_012475747.1	LOC105791964	B456_004G188300	0	50360617	50365913	2365	553	4	15	14	61995.85	6.23	46.72-unstable	68.05	-0.704	Nuclear	Reverse
54	GrPRR3	XP_012446241.1	LOC105769855	B456_009G103700	0	7501708	7508343	2680	698	9	13	12	76645.02	6.19	46.68-unstable	63.68	-0.699	Nuclear	Reverse
55	GrPRR5.1	XP_012460167.1	LOC105780403	B456_012G001000	0	123376	127728	2653	705	12	8	7	78569.28	8.58	55.44-unstable	67.48	-0.699	Nuclear	Forward
56	GrPRR5.2	XP_012488628.1	LOC105801851	B456_007G017100	0	1308225	1312527	2356	635	7	8	7	70246.32	6.72	51.47-unstable	66.35	-0.701	Nuclear	Forward
57	GrPRR7.1	XP_012435203.1	LOC105761814	B456_007G374800	0	60664640	60670196	3091	747	7	11	10	80800.81	7.88	44.12-unstable	68.93	-0.689	Nuclear	Forward
58	GrPRR7.2	XP_012459739.1	LOC105780145	B456_012G092600	0	16303753	16310678	2936	750	12	10	9	81705.02	7.10	41.54-unstable	72.64	-0.717	Nuclear	Reverse
59	GrPRR9	XP_012490182.1	LOC105802847	B456_007G113000	0	8758521	8763108	2589	652	7	9	8	72804.40	5.40	51.07-unstable	71.47	-0.638	Nuclear	Forward

### Phylogenetic analysis

The purpose of this work was to investigate the evolutionary and phylogenetic relationships of *G. raimondii’s* TCS genes with their counterparts; a comparative tree was generated of target specie (*G. raimondii*), *A. thaliana*, *O. sativa*, *C. arietinum, S. bicolor and G. max* (**Supplementary Figure 1**). TCS elements were classified as HKs, HPs and RRs. HKs (Histidine kinases) were categorized into three groups: AHKs, ethylene receptors and phytochromes. AHK has six subfamilies named AHK2, AHK3, AHK4, AHK1, AHK5 and CKl1. First three subfamilies are also known as cytokinin receptors because they have additional cytokinin binding CHASE domain. These cytokinin receptors (GrHK2.1, GrHK2.2, GrHK3.1, GrHK3.2, GrHK4.1, and GrHK4.2) were discovered in *G. raimondii* which are similar to the AHK4, AHK3, and AHK2 found in *A. thaliana*. On the basis of the phylogenetic tree, these receptors were orthologues of *A. thaliana’HKs.* In *A. thaliana*, cytokinin receptors responsible for variety of functions including stress responsive, propagation of root and shoot, fruit ripeness and softness, and it is possible that *G. raimondii’s* cytokinin receptors have similar features.

AHK1 is a TM protein that participates in osmo-sensing and is highly exhibited in *A. thaliana* roots when exposed to salinity stress. In *G. raimondii*, GrHK5.1 and GrHK5.2 are orthologous of AHK5 (also known as CKI2). GrHK1, GrCKI1.1 and GrCKI1.2 are orthologous of AHK1 and CKI1 respectively. Phylogenetic study revealed that identified ethylene receptors GrETR1.1, GrETR1.2, GrERS1.1 and GrERS1.2 are exact homologous to AETR1 and AERS1. GrETR2 and GrERS2 are paralogues, they have orthologous relationship to AtETR2 and AtERS2. On the other hand, GrEIN4 is orthologous of *A. thaliana* EIN4.

Plant growth and development are controlled by phytochromes. These are light responsive elements (also referred to as photoreceptors). These receptors are responsible for signal transmission in model plant *A. thaliana*. In target specie, GrPHYA1, GrPHYA2, GrPHYB, GrPHYC and GrPHYE perform the same functions as in *A. thaliana*. *G. raimondii* has eight HPs, all of which are closely related to *A. thaliana* and *O. sativa* in terms of phylogeny. The phylogenetic relations between these HPs and their counterparts (*A. thaliana*) were used to group them. GrHP1.1 and GrHP1.2 were classified as AHP1-like. GrHP3 and GrHP5 were named AHP3-like and AHP5-like. GrHP4.1 and GrHP4.2 were classified as AHP4-like. GrHP6.1 and GrHP6.1 were grouped as AHP6-like HPs.

For the phylogenetic study of response regulators, protein sequences from different species such as *A. thaliana, G. max, S. bicolor, C. arietinum, O. sativa* and *G. raimondii* were employed. There are 4 sub-types of RR family (type-A, type-B, type-C and PRRs) on the basis of conserved domains. These individuals control the final reactions to environmental challenges. Divergent RRs are not regarded as real RRs because they don’t have the DDK (conserved motif). Because GrRRs are closely related to *A. thaliana* and *O. sativa* in terms of phylogeny. So, it was determined that they are real RRs. In plants, type-A RR is regarded as new RR family because it is not present in unicellular algae. They have been proposed to conduct several novel activities in plants. The REC domain as well as the DDK motif are present in RRs which are required for phosphate group acceptance. Cytokinin is the primary stimulator for type-A RRs, and type-B RRs play a role in cytokinin induction.

### Analysis of gene structure and conserved motifs

**Figure 3:**
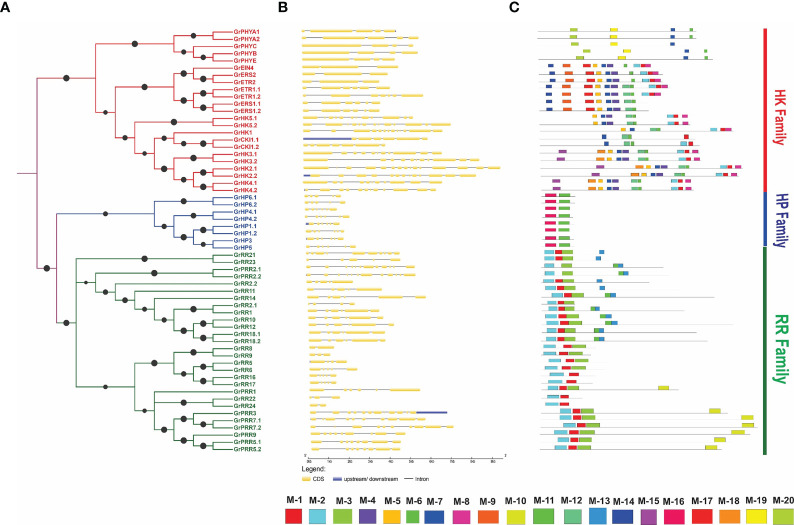
Structural features of GrTCS. **(A)** Phylogeny of GrTCS genes. **(B)** Shows the GrTCS gene structure assessment. In this section, introns, exons and UTRs are denoted by black lines, yellow and blue boxes, respectively. **(C)** The GrTCS conserved motifs are shown in this final section. Each specific color box represents a specific motif.

The intron-exon pattern of genes is an important characteristic that offers knowledge about the gene’s various functions. So, we further investigated the intron-exon pattern of the GrTCS genes. The findings indicate that cytokinin receptors (GrHK1-GrHK5.2) have 11-17 exons and 10-16 introns (**Figures 3A, B**), which are comparable to *A. thaliana* cytokinin receptors (exons from 11 to 14 and introns from 10 to 13). AtCKl1 has nine introns and nine exons, whereas GrCKI1.1 and GrCKI1.2 each have six introns and seven exons. Ethylene responsive elements GrETR1.1, GrERS1.1 and GrERS1.2 were found to have five introns and six exon while GrETR2, GrERS2 and GrEIN4 have two introns and three exons. GrETR1.2 was shown to have eight exons and seven introns. This intron-exon pattern is identical to the intron-exon organisation of *A. thaliana* ethylene sensors. GrPHYA1, GrPHYA2, GrPHYB, GrPHYE were discovered to contain four introns and five exons among *G. raimondii* phytochrome family. GrPHYC was shown to contain four exons and three exons. The HP candidates GrHP1.1 and GrHP3 have 7 exons and 6 introns, GrHP1.2, GrHP4.1, GrHP4.2 and GrHP5 have 6 exons and 5 introns while GrHP6.1 and GrHP6.2 have 5 exons and 4 introns.

The number of introns in the *G. raimondii* response regulators ranged from one to fourteen while the exons number ranged from two to fifteen. GrPRR2.2 has greatest number of introns-exons (14 and 15 respectively) which is more than the introns-exons number in *A. thaliana* RRs. These findings revealed that identified members have a similar intron-exon pattern with their phylogenetic ties. The MEME tool was then used to estimate the twenty conserved motifs in identified TCS elements (**Figure 3C**). *G. raimondii* cytokinin receptors have 18, 17, 15, 8, and 2 conserved motifs, which are likewise seen in *A. thaliana*. They also have 4.5, 7, 11 and 12 additional conserved motifs. GrCKI1.1 has 2, 7, 11, 12 and 17 conserved motifs. The same motifs found in GrCKI1.2 (except 2). From these conserved motifs 2 and 17 similar to AtCKI1. Ethylene receptors have conserved motifs that are similar to cytokinin receptors and with the 1 and 14 additional motifs. *G. raimondii* phytochromes have four conserved motifs (3, 11, 14 and 19). Except for motif 3, these members share three motifs with *A. thaliana*: 11, 14, and 19. There were only two conserved motifs (11 and 16) in GrHPs.

All type-A response regulators have 3, 2 and 1 conserved motifs; GrRR16 and GrRR17 do not have 3 conserved motifs. Type-B response regulators have similar motifs to type-A, but they include two additional conserved motifs (13 and 6). Two conserved motifs (1 and 2) are found in Type-C RRs (GrRR22 and GrRR24). The PRRs have similar motifs to type-A response regulators but with the 6, 13 and 19 additional motifs. Similar motifs present in same families signify that their functions or sequences are not more diverse. Consequently, two component system is conserved according to evolutionary studies.

### Genomic distribution, gene duplication, and syntenic analysis of the GrTCS elements

A syntenic study was used to identify the chromosomal gene locations and duplicated occurrences of the GrTCS elements in order to investigate their genomic distribution. All 59 detected TCS elements in *G. raimondii* are located at one to thirteen chromosomes. There are a large number of GrTCS genes (12) on chromosome 7, whereas chromosomes 1 and 2 have just two genes. *G. raimondii* HK proteins are dispersed on all thirteen chromosomes except chromosomes 4, 10 and 12. The HP proteins are found on chromosomes 6, 7, 9, 10 and 11. The RR proteins are present on all thirteen chromosomes (**Supplementary Figure 2**).

Duplication events of *G. raimondii* TCS gene families were investigated. Duplication events offer essential material for development and assist in genome analysis to investigate the evolution of novel genes. Many plants have an increase in the frequency of duplication events (tandem or segmental). We identified syntenic homologues (5 pairs) in the *G. raimondii* genome for duplication events. It revealed that *GrHK3.1/GrHK3.2, GrETR1.1/GrETR1.2, GrERS1.1/GrERS1.2, GrPHYA1/GrPHYA2* and *GrHP6.1/GrHP6.2* are genes with the segmental duplication. Expansion of *G. raimondii*’s TCS proteins occurs mostly by segmental duplications. This pattern is similar to several plants like *A. thaliana* and *G. max*. We computed both synonymous and non synonymous substitutions (Ks and Ka) of GrTCS proteins duplications, as well as their ratio (Ka/Ks). The Ks values have been employed to estimate the time required for duplication. GrTCSs Segment duplicates had Ks ranging 1.483-3.968. As a result, the diverging period spanned 113.0182927-302.4085366 MYA (**Table 3**).

**Table 3:** *G. raimondii* duplicated gene pairs displaying the computing Ka/Ks, Ka and Ks values and diverging time.

Duplicated gene pairs		Chr	Ks	Ka	Ka/Ks	Type	Time
GrERS1.1	GrERS1.2	8,7	1.483	2.182	1.47133801	Segmental	113.0182927
GrETR1.1	GrETR1.2	2,9	1.633	2.011	1.23160796	Segmental	124.4283537
GrHK3.1	GrHK3.2	5,8	1.947	2.456	1.26195736	Segmental	148.3612805
GrPHYA1	GrPHYA2	13,7	2.009	3.482	1.7334827	Segmental	153.0868902
GrHP6.1	GrHP6.2	6,10	3.968	2.374	0.5983214	Segmental	302.4085366

### Putative promoter analysis of GrTCS genes

The Promoter region of *G. raimondii’s* TCS elements was studied to anticipate the *cis*-elements and obtain a better knowledge of their functions and transcriptional control. Numerous abiotic responsive and hormonal related *cis*-elements were discovered, including the TATA box and CAAT box which were found in nearly all GrTCS genes. In 26 GrTCS, TGACG and CGTCA motifs (MeJa responsive elements) have been discovered. ABRE element is involved in the abscisic acid response and was discovered in 36 of the 59 GrTCS genes. The ATCT, ATC, TCCC, AT1, GATA, GT1, TCT, CAG, and ACA motifs, as well as the I-box, Box 4, G-Box, G-box, Box-II, AE-box, MRE, chs-cMA1a, and chs-cMA2a *cis*-elements, were all connected to light responsiveness in GrTCS. The O2-site was found in 11 GrTCS genes and was associated with zein metabolism control.

GrTCS promoter regions also contained a TCA-element, which was associated with salicylic acid responsiveness. The GARE motif, P, and TATC boxes were found in 17 genes and were determined to be responsible for gibberellin responsiveness. In 39 GrTCS genes, ARE *cis*-element was present and revealed to be responsible for anaerobic induction. GrTCS found two *cis*-elements (TGA element and AuxRR-core) linked to auxin responsiveness. LTR and MBS elements were discovered in 12 GrTCS genes that respond to low temperature and drought respectively (**Supplementary Figure 3**). These findings suggest that GrTCS proteins serve key role in signaling pathways during activities of plant growth and development, as well as they were responding to different abiotic stresses.

### Differential expression pattern of the TCS genes in *Gossypium raimondii*

The *G. raimondii* is known to be more tolerant to salinity stress and cold stress than the cultivated cotton species *G. hirsutum.* Therefore, understanding the mechanisms of stress response in *G. raimondii* can provide insights into improving stress tolerance in cultivated cotton. Transcriptome analysis can identify genes and pathways that are differentially regulated under combined stress conditions, and provide a better understanding of how plants respond to stress at the molecular level. This information can be used to identify key genes and pathways involved in stress response and to develop molecular markers for selecting stress-tolerant cotton cultivars. Furthermore, *G. raimondii* is a valuable model system for studying the molecular basis of stress tolerance in plants. As a diploid species with a smaller genome size than *G. hirsutum*, it is easier to study the functional genomics of *G. raimondii*. Transcriptome analysis can provide a foundation for further functional analysis of genes and pathways involved in stress tolerance in cotton.

**Figure 4:**
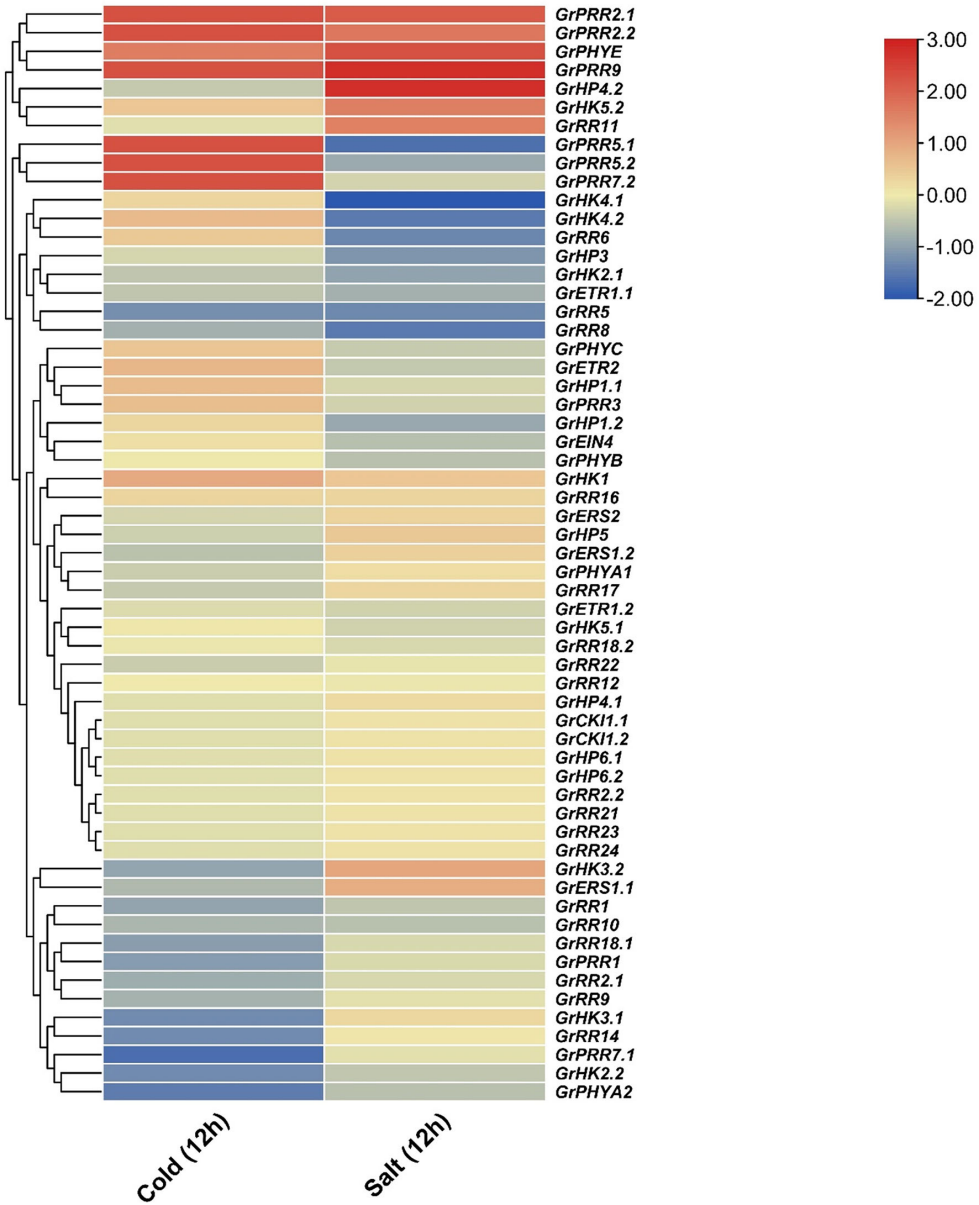
RNA-seq based expression profile of GrTCS genes. The heatmap is graphical representation of GrTCSs response against salt and cold stresses. Dark red, blue and light yellow color indicates the up, down regulation and non-responsive expression, respectively.

The NCBI server’s Sequence Read Archive (SRA) Database was utilized to obtain publically accessible RNA sequencing data in order to investigate the differential expression of 59 identified GrTCS genes in response to cold and salt stresses (**Supplementary Table 2**). It was observed that *GrPRR2.1, GrPRR2.2, GrPRR9, GrPHYE*, and *GrHK1* were highly upregulated in reponse to both stresses (salt and cold). On the contrary, *GrRR1*, *GrRR5, GrRR8*, and *GrRR10* were highly downregulated in response to both stresses. Meanwhile, *GrPRR5.1, GrPRR5.2*, and *GrPRR7.2* were upregulated in response to cold stress and downregulated in response to salt stress. Similarly, *GrHP4.2, GrHK5.2*, and *GrRR11* were upregulated in response to salt sress and downregulated in response to cold stress (**Figure 4**). These findings suggest that GrTCS genes may serve key role in responding to different abiotic stresses.

**Figure 5:**
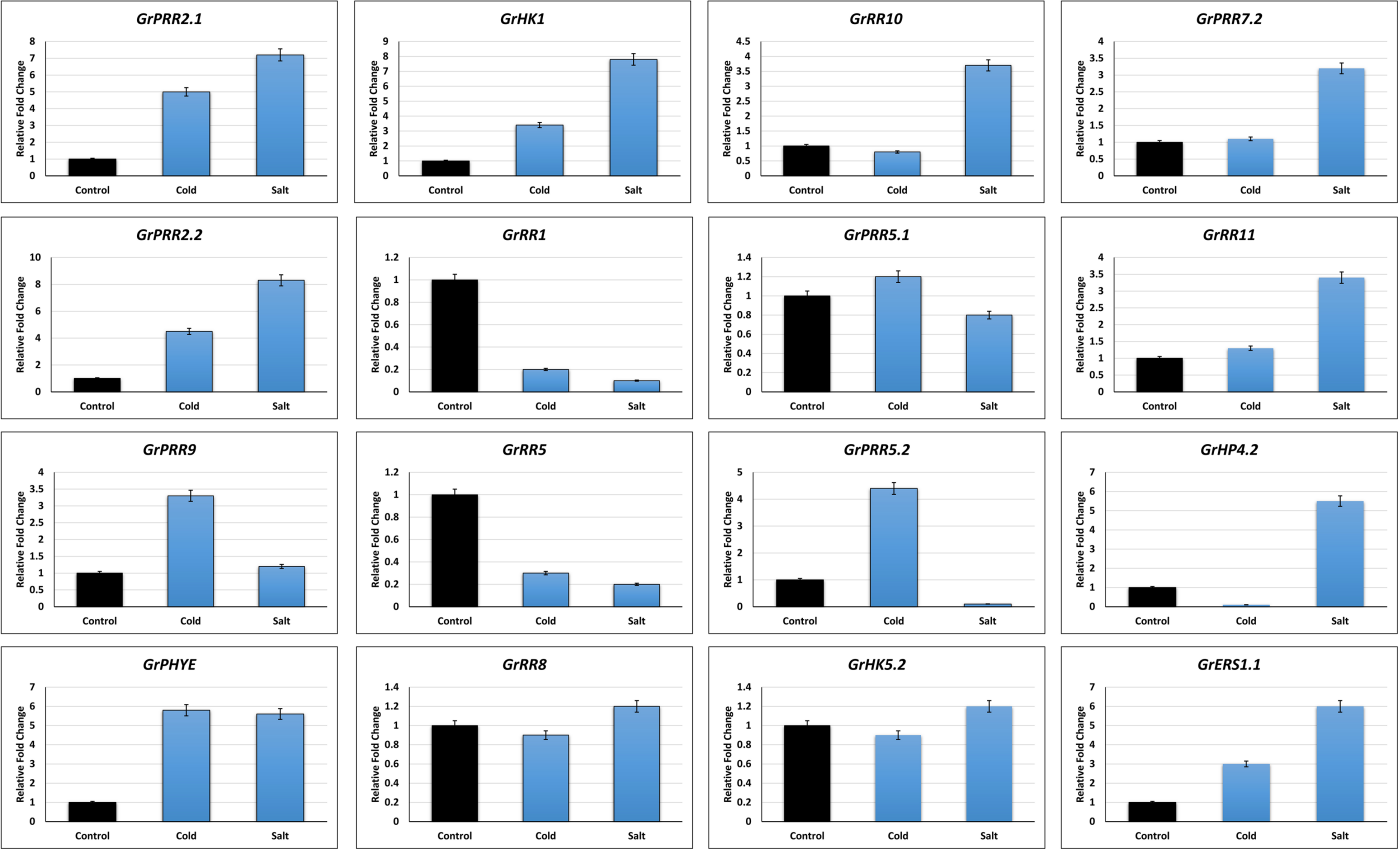
Relative Real-time qPCR based expression profiling of GrTCs members.

**Figure 6:**
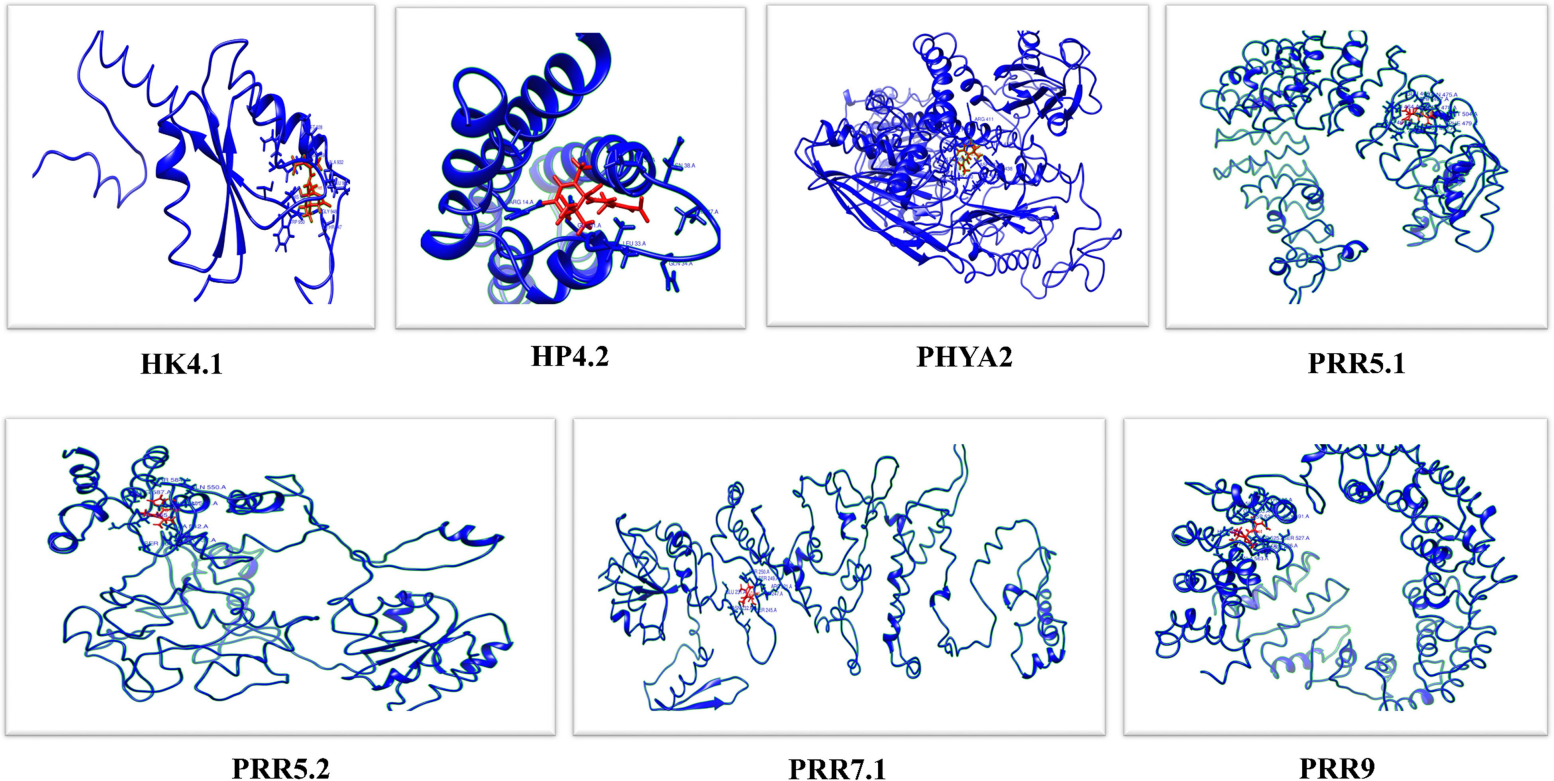
Docking of seven GrTCS proteins with abcisic acid (plant-hormone). The protein structures are indicated by dark blue color, while the ligand is indicated by dark red color (abcisic acid). The conserved residues are likewise labeled in blue in this figure.

A relative real-time qPCR analysis was carried out to confirm the expression profiles of highly differentially expressed genes obtained from RNA-seq data (**Figure 5**). The expression profiles correlated with RNA-seq data with some variations. The *GrHK1, GrPHYE, GrERS1.1, GrPRR2.1* and *GrPRR2.2* were significantly upregulated in response to both stresses. Similarly, *GrRR1* and *GrRR5* were downregulated in response to both stresses. The transcripts of *GrPRR9, GrPRR5.2* were upregulated and *GrHP4.2, GrRR10, GrRR11, GrPRR7.2* were downregulated in response to cold stress and vice versa for salt stress. Interestingly, no variation of expression was observed for *GrHK5.2, GrRR8* and *GrPRR5.1* for both stresses (**Figure 6**).

### 3D structure prediction of differentially expressed GrTCS genes

Proteins are tough to explain because these are complicated chemical structures that include a vast range of winding topologies as well as atoms that may take on a variety of different configurations. During this investigation, some accurate predictions about the 3D-structures of 7 different TCS proteins including GrHK4.1, GrHP4.2, GrPHYA2, GrPRR5.1, GrPRR5.2, GrPRR7.1 and GrPRR9 were made (**Supplementary Figure 4**). These genes were selected for further analysis based on whether they showed high upregulation, downregulation, or no expression at all in response to cold or salt stress. GrHK4.1 expression highly decreased in response to salt stress and increased to some extent against cold stress. The expression of GrHP4.2 was shown to be significantly upregulated in reaction to salt stress, whereas it was found to be almost decreased in response to cold stress. GrPHYA2 expression was found to be significantly reduced in response to cold stress, and it was also shown to be reduced, although to a lesser level, in response to salt stress. In reaction to salt stress, GrPRR5.1 expression was found to be significantly reduced, but it was shown to be highly elevated in response to cold stress. GrPRR5.2 expression highly increased in response to cold and decreased to some extent against salt stress. The expression of GrPRR7.1 was shown to significantly decrease in reaction to cold and has almost zero expression in response to salt stress. In reaction to both salt and cold stresses, GrPRR9 expression was found to be significantly elevated. The final structures exhibited favorable stereochemical properties. GrHK4.1, GrHP4.2, GrPHYA2, GrPRR5.1, GrPRR5.2, GrPRR7.1, and GRPRR9 genes exhibited strong overall quality. In the predicted models, helices are shown in yellow, whereas sheets and strands are shown in green. Proteins that are members of the same family often possess a structural symmetry that is quite identical. The structures of the proteins belonging to the RR family are almost identical.

### Docking of differentially expressed GrTCS genes

The decision to use abscisic acid (ABA) as the ligand for docking studies with TCS genes in *G. raimondii* was based on the known role of ABA in plant responses to abiotic stress, particularly salinity and cold stress. ABA is a key phytohormone involved in regulating plant responses to environmental stresses, including water deficit, salt stress, and cold stress. ABA signaling pathways are known to interact with two-component systems in plants, making ABA a relevant ligand for studying these interactions. Furthermore, the use of ABA as the ligand in docking studies was motivated by the goal of understanding the specific role of ABA in regulating the responses of TCS genes to salinity and cold stress. By using ABA as the ligand, the study aimed to identify specific interactions between ABA and TCS genes that are important for mediating stress responses. It is also important to note that the selection of ABA as the ligand does not preclude the possibility of studying other plant hormones and their interactions with TCS genes in future research. However, by focusing on ABA in this study, we were able to gain a more detailed understanding of the specific role of ABA in regulating stress responses in *G. raimondii*.

Molecular Docking (MD) of abscisic acid was conducted with seven different GrTCS proteins to investigate the binding interactions (**Figure 6**). The findings of the Molecular Docking demonstrated that all of the GrTCSs had a strong capacity to bind. Docking complexes possess the best score and RMSD value possible, which is a range from -13.3339338 (GrPRR5.1) to -6.74946165 (GrPRR9) for their S-score and from 1.04909098 (GrPRR5.1) to 2.34853554 (GrPRR9) for their RMSD. GrHK4.1’s conserved residues include MET 928, ILE 925, GLN 899, LEU 900, TRP 950, HIS 902, GLY 948, THR 947, GLU 929, and ALA 932. The bond lengths of these residues vary from 1.686 Å (THR 947) to 3.294 Å (GLN 899). GrHP4.2’s conserved residues were ARG 14, GLU 31, LEU 33, GLN 34, ASN 38, THR 37, and PRO 39 and bond lengths of these residues ranged from 1.604 Å to 3.201 Å. GrPHYA2 possessed 12 conserved residues including ARG 411, THR 412, ASP 437, VAL 439, MET 436, LYS 440, LEU 959, VAL 957, LEU 438, ASN 408, GLY 955, and ASP 958 and their bond lengths ranged from 1.482 Å to 2.863 Å. GrPRR5.1 possessed conserved residues including GLN 475, LEU 464, LEU 478, PHE 479, PRO 482, VAL 462, THR 461, MET 504, THR 467 and GLN 466. The bond lengths of these residues vary from 1.480 Å to 2.758 Å. GrPRR5.2 possessed conserved residues including GLN 550, THR 584, VAL 565, ARG 587, SER 560, ASN 547, GLU 551, ALA 542 and TYR 537. Their bond lengths ranged from 1.260 Å to 2.951 Å. GrPRR7.1’s conserved residues include SER 250, SER 249, ARG 321, GLU 231, THR 247, SER 245 and ASN 232. The bond lengths of these residues vary from 1.306 Å to 3.318 Å. GrPRR9 contained 12 conserved residues such as TYR 490, GLY 491, SER 527, GLN 526, GLN 503, ASP 524, LEU 532, ALA 563, HIS 539, THR 559, TYR 525, and GLN 506. Their bond lengths ranged from 1.502 Å to 2.669 Å (**Supplementary Table 1**).

## Discussion

The growth and production of plants can be hindered due to their susceptibility to various biotic and abiotic stressors. Plants with their immobility are not able to resist these circumstances. As a result, they have developed several signalling systems to survive (Gupta et al., 2020; Rasul et al., 2017). The two component system (TCS) is essential for signalling, and contributes to the development of plants with enhanced characteristics like stress tolerance (Gupta et al., 2020). Members of TCS have been studied in several plant species, but in target plant (*G. raimondii*), the diversification of TCS gene family is never revealed. We used multiple analyses to identify and validate TCS genes in the current study.

TCS genes have been found in several plant species, such as *T. aestivum, A. thaliana, O. sativa, S. bicolor, G.max, C. arietinum*, and *C. melo L*. (Liu et al., 2020) *G. raimondii* has 59 genes of TCS in its genome that is more than TCS members of *A. thaliana, O. sativa*, and *S. bicolor* (47, 37 and 37, respectively) but less than *G. max, T. aestivum, C. arietinum* and *C. melo L.* (98, 62, 51 and 51, respectively). TCS genes are scattered on various chromosomes in *A. thaliana*, *Brassica rapa*, *Zizania latifolia*, and *Cucumis melo* L., and these genes are duplicated segmentally and tandemly. The majority of segmentally duplicated genes have been detected in *B. rapa* (Liu et al., 2014). There is one tandem incidence in *Cucumis sativus L* (He et al., 2016b). *Citrullus lanatus*, on the other hand, had one tandem and two segmental occurrences. There are 19 duplicated genes in *Z. latifolia* (He et al., 2020).

The five gene pairs (GrHK3.1/GrHK3.2, GrETR1.1/GrETR1.2, GrERS1.1/GrERS1.2, GrPHYA1/GrPHYA2, and GrHP6.1/GrHP6.2) represent segmental duplication in *G. raimondii*, leading to an enlargement of the GrTCS gene family. Similar segmental duplications have also been observed in other plants such as *B. rapa* (Liu et al., 2014), *C. arietinum* (Ahmad et al., 2020), and *A. thaliana* (Du et al., 2007). In *B. rapa*, 61 out of 85 TCS candidate genes were found to be segmentally duplicated. *A. thaliana* and *C. arietinum* have segmental duplication rates of approximately 35.7% and 55.6%, respectively. *Solanum lycopersicum* has both segmental and tandem duplications, with synonymous substitutions of 0.6-0.79 and 46-60 for the two types, respectively (He et al., 2016a). The divergence time for segmentally duplicated genes in *G. raimondii* ranges from 113.01 to 302.40 million years ago, whereas the divergence time for tandemly duplicated genes in *S. lycopersicum* ranges from 5.96 to 26.55 million years ago. Tandem duplication appears to be more common than segmental duplication, as it may confer a greater ability to respond to different stressors (Firon et al., 2012).

According to phylogenetic analysis, identified genes in the target plant were grouped into separate families and subfamilies, as in other plants with TCS systems (Arabidopsis, Rice, Sorghum bicolor, and Tomato). Candidates belonging to the three subgroups HK, RR, and HPs are found in all of the plants listed above. The functional domains that are preserved in *G. raimondii* and *A. thaliana* were used to classify the subgroups. AtCKI1 is referred to as a Hybrid HK (L) because of its role in cytokinin transduction (Ishida et al., 2009). GrCKI1.1 and GrCKI1.2 performed a similar function. The CKI2 subgroup includes two genuine HKs, GrHK5.1 and GrHK5.2. *A. thaliana* response regulators are characterized by the presence of a REC domain. Response regulators have a comparable domain in the target plant *G. raimondii*. The presence of a conserved domain (CHASE) places *C. arietinum* cytokinin receptors in a distinct clade in phylogenetic analysis. Likewise, *G. raimondii* cytokinin receptors have an extra TM domain and the CHASE domain.

The Promoter region of the *G. raimondii’s* TCS elements was studied to identify the *cis*-elements and aided in the identification of switches engaged in the regulation of transcriptional activity of downstream candidates. The existence of many light, salicylic acid, abscisic acid, drought, hormone, stress and defense-related, low-temperature, and auxin-responsive switches were shown by our recent research findings(Ahmad et al., 2020; Zameer et al., 2021). In many other plants, such switches have already been discovered in their promoter regions. Like light, ABRE, gibberellin, and hormone responsiveness switches present in histidine kinase family of bananas (Dhar et al., 2019), while response regulators have many stress related switches. A considerable number of *cis*-elements such as drought and abscisic acid responsiveness elements were discovered in *C. sativus L.* and *C. lanatus*. Aside from abiotic stresses, In *B. rapa*, response regulators of type-A possess an additional binding site known as GARP, which may contribute to an increase in available binding sites for type-B response regulators and lead to transcriptional boosting (Liu et al., 2014). This may contribute to the availability of binding sites for type-B response regulators, leading to transcriptional boosting. Type-A response regulators were discovered to be somewhat dependent on type-B response regulators. As a result, The TCS gene family has been found to respond to both biotic and abiotic stresses, including hormonal stress.

Plant growth and development may be influenced by abiotic stressors including heat, water, and salinity. In plants, TCS genes have a function in managing the response against several abiotic challenges. The expression study of this gene family may lead to a better understanding of its role in environmental adaptation. AtHK1 functions as a strong activator against dehydration and salinity stressors in *A. thaliana* (Tran et al., 2007). In the present investigation, it was found that the GrTCS gene family has different expressions in different tissues. *GrPHYE, GrHP4.2, GrPRR2.1*, and *GrPRR9* were shown to be increased in leaves against salt stress. Numerous RR candidates and only one HK candidate such as *GrPRR2.1, GrPRR2.2, GrPRR5.1, GrPRR5.2, GrPRR7.2, GrPRR9*, and *GrPHYE* were increased in response to cold stress. However, *GrHK4.1, GrHK4.2, GrPRR5.1*, and *GrRR8* all had lower expression levels against salinity. Several HK and RR candidates, including *GrHK2.2, GrHK3.2, GrPHYA2, GrRR5, GrRR14*, and *GrPRR7.1* were discovered to be decreased in response to cold stress. Most HP candidates showed no changes in expression when treated to cold stress. Chickpea members such as CarHK2, 3 and 4 have been exhibited in most tissues. Two potential Chickpea HK1 and HK5 genes have been found in the plant’s pods and shoots. The majority of expression of genes belonging to the CarRR family was found in floral buds. In a similar manner, candidates for rice’s HK seemed to be detected in the leaf and roots whereas HP candidates have been detected in the leaves. Response regulators have been discovered in spikelets, leaf, and stems. The melons with the greatest level of RR expressions provide evidence that these elements play a significant role in the root cytokinin signalling process (Liu et al., 2020). The Chinese cabbage had the same findings as the previous study (Liu et al., 2014).

One of the most significant challenges facing contemporary agriculture is the impact of abiotic stressors, including salinity and water deficit. A growing body of data suggests that TCS elements play a crucial role in responding to these stressors. During a recent study, 59 GrTCS elements were identified, and it was revealed that certain genes were up-regulated or down-regulated in response to cold and salt stress. Thirty-six of the 59 GrTCS elements were affected by cold stress, while 26 were affected by salt stress. The expression profile of TCS elements has a significant impact on their response to abiotic stressors.

## Conclusion

In current study, we identified 59 candidates for the TCS, which were categorized into three subfamilies. We conducted in-depth analysis of protein classifications, predictions of domains, evolutionary connections, gene architectures, dispersion of genes on different chromosomes, and occurrences of duplicate genes. Our results indicate that TCS has highly conserved sequences as well as specialized domains that can play important roles in executing various functions. We found that the phylogenetic link between GrTCS elements and TCS genes from other plant species is more closely related. Notably, we observed that GrHK candidates were duplicated segmentally, which led to the duplication of genes and ultimately to their extension. Differential expression in response to cold and salt stresses revealed that *GrPRR2.1, GrPRR2.2, GrPRR9, GrPHYE*, and *GrHK1* were highly upregulated, while *GrRR1, GrRR5, GrRR8*, and *GrRR10* were highly downregulated in response to both stresses. Furthermore, *GrPRR5.1, GrPRR5.2*, and *GrPRR7.2* were upregulated in response to cold stress and downregulated in response to salt stress, whereas *GrHP4.2, GrHK5.2*, and *GrRR11* were upregulated in response to salt stress and downregulated in response to cold stress. Real-time qPCR analysis confirmed the RNA-seq data, indicating the potential role of GrTCS genes in responding to abiotic stresses. These findings provide a better understanding of the signaling cascades and the means by which *G. raimondii* might improve its capacity to endure the effects of different biotic and abiotic stressors.

## Data availability statement

The datasets presented in this study can be found in online repositories. The names of the repository/repositories and accession number(s) can be found in the article/**Supplementary Material**.

## Author contributions

FA, AR, SQ: investigation, data analysis, writing-original draft preparation, writing-review & editing. MU-R: methodology. MR, MS, DH: participated in the experiment, and data analysis. visualization. NA: Methodology. FA: conceptualization. All authors contributed to the article and approved the submitted version.

## Supplementary material

Supplementary Figure 1 Cummulative phylogenetic tree of GrTCS. proteins from *A. thaliana, S. bicolor, O. sativa, C. arietinum, G. raimondii and G. max*. In MEGA X, neighbor-joining methodology of 1000 bootstraps was utilized to create the tree. Particular colors are used to symbolize each species and subgroups. Candidates from GrTCS are denoted by a dashed-lines and dark blue color.

Supplementary Figure 2 The synteny of GrTCS members. The chromosomal positions are indicated by the genes on various bar blocks in circle. The five duplicated pairs are represented by color arcs in red, green, yellow, blue, and pink.

Supplementary Figure 3 GrTCS’ promoter evaluation. *cis*-regulatory components are graphically shown. These elements indicated by distinct colors, while the numbers in the bars show the frequency of elements.

Supplementary Figure 4 Final 3D structures of seven GrTCS proteins. Different colours were used in these models’ predictions to represent various sheets, helical structures and domains.
