# Supplementary figures and images for: Omics-assisted characterization of two-component system genes from Gossypium Raimondii in response to salinity and molecular interaction with abscisic acid
Source: Front Plant Sci. 2023 Mar 31;14:1138048. doi: 10.3389/fpls.2023.1138048 (PMC10102465; doi:10.3389/fpls.2023.1138048)

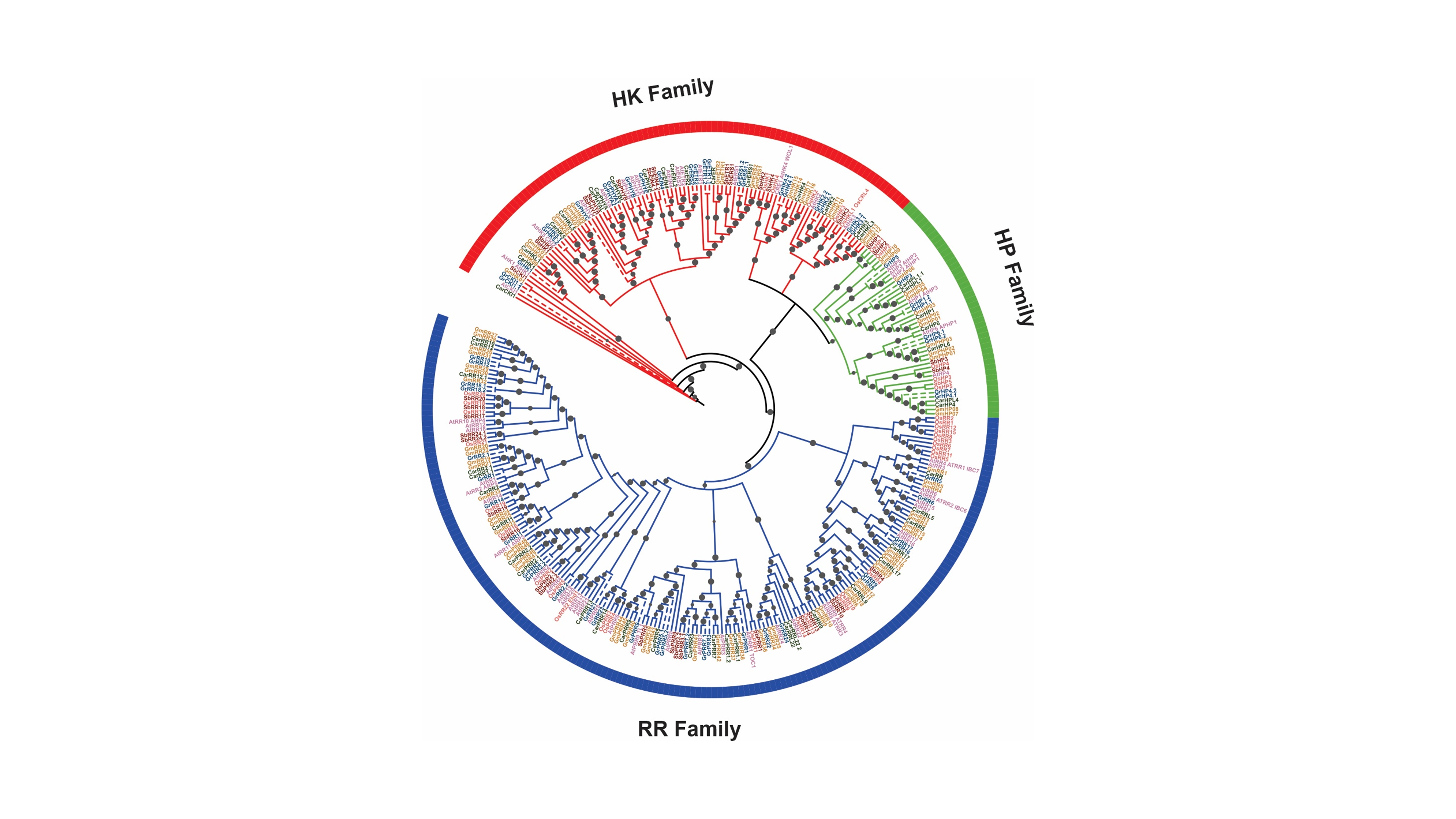

Supplement: Supplementary Figure 1 — Cummulative phylogenetic tree of GrTCS. proteins from A. thaliana, S. bicolor, O. sativa, C. arietinum, G. raimondii and G. max. In MEGA X, neighbor-joining methodology of 1000 bootstraps was utilized to create the tree. Particular colors are used to symbolize each species and subgroups. Candidates from GrTCS are denoted by a dashed-lines and dark blue color. [file Image_1.jpeg]

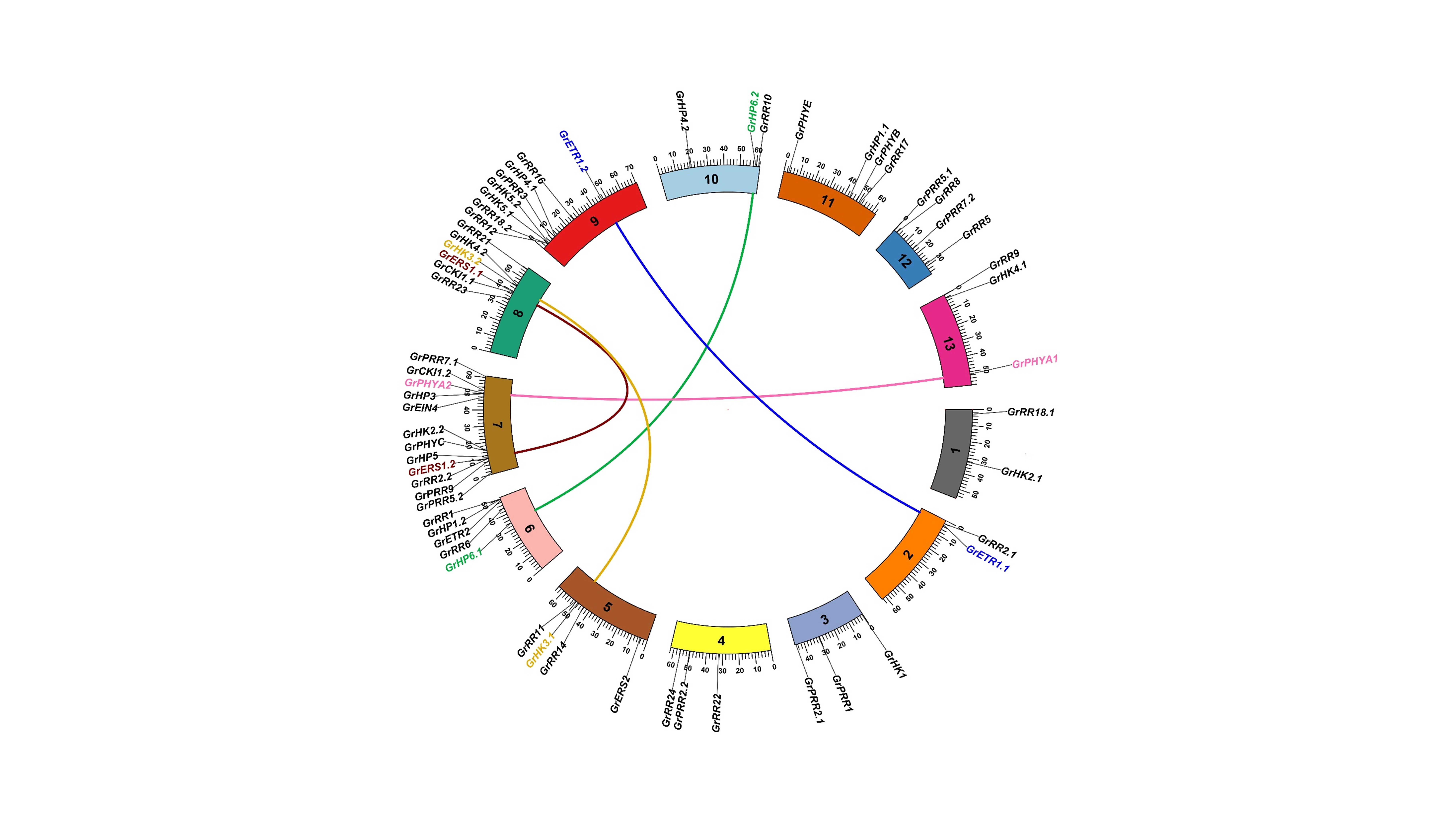

Supplement: Supplementary Figure 2 — The synteny of GrTCS members. The chromosomal positions are indicated by the genes on various bar blocks in circle. The five duplicated pairs are represented by color arcs in red, green, yellow, blue, and pink. [file Image_2.jpeg]

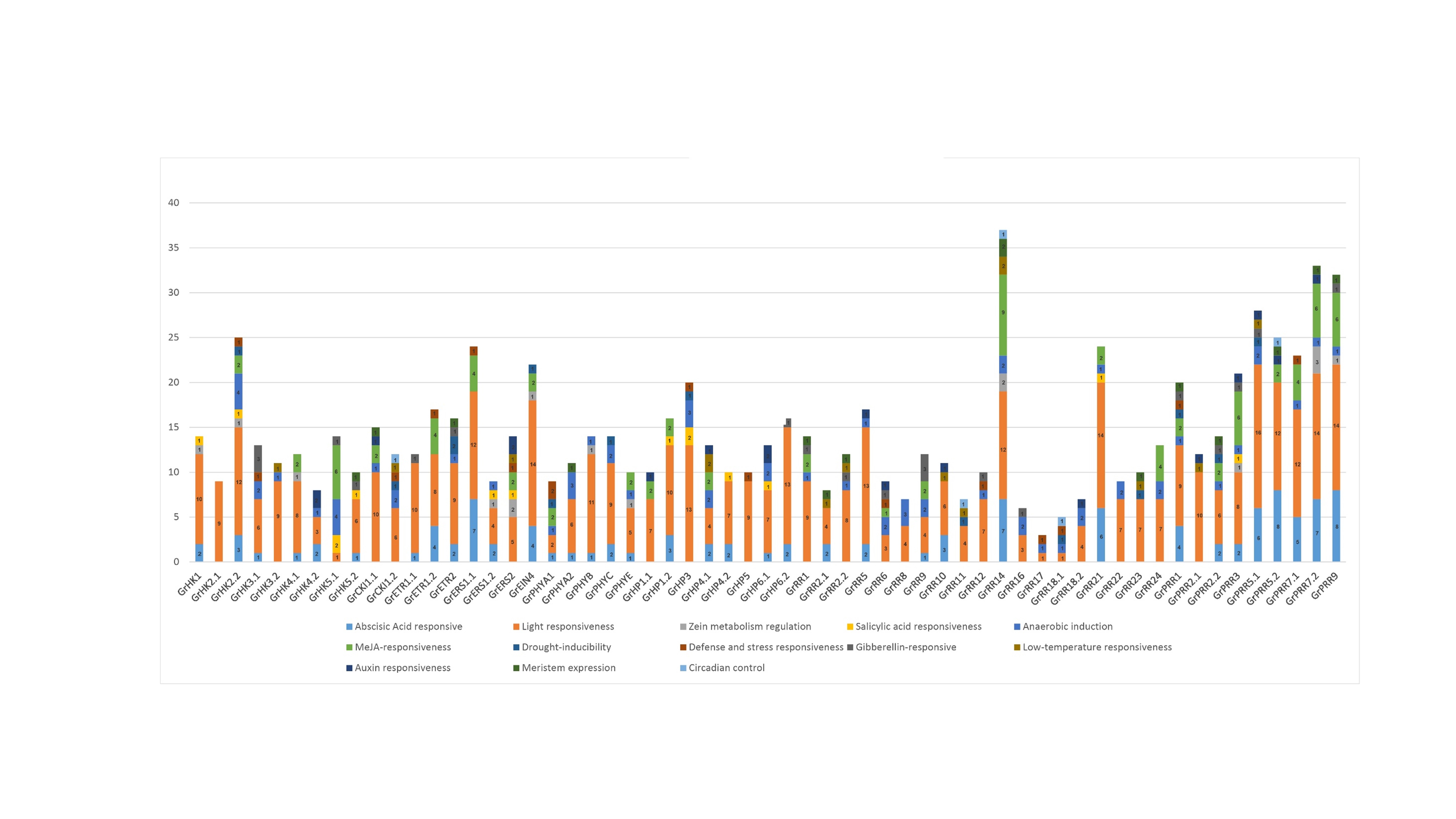

Supplement: Supplementary Figure 3 — GrTCS’ promoter evaluation. cis-regulatory components are graphically shown. These elements indicated by distinct colors, while the numbers in the bars show the frequency of elements. [file Image_3.jpeg]

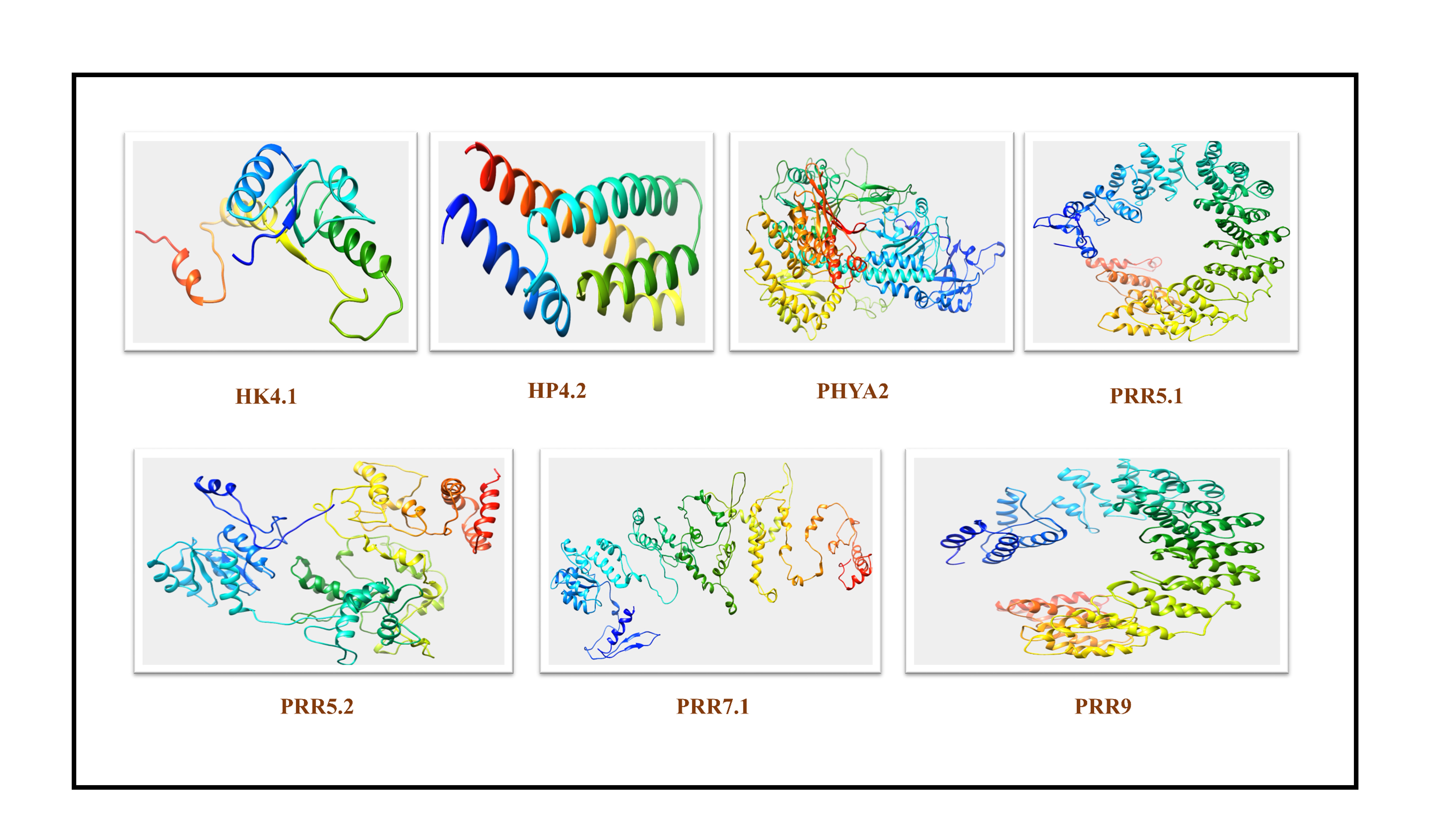

Supplement: Supplementary Figure 4 — Final 3D structures of seven GrTCS proteins. Different colours were used in these models’ predictions to represent various sheets, helical structures and domains. [file Image_4.jpeg]
