# Supplementary material for: Omics-assisted characterization of two-component system genes from Gossypium Raimondii in response to salinity and molecular interaction with abscisic acid
Source: Front Plant Sci. 2023 Mar 31;14:1138048. doi: 10.3389/fpls.2023.1138048 (PMC10102465; doi:10.3389/fpls.2023.1138048)
Supplement: Supplementary file 5 [file Table_1.docx]

Supplementary table 1. Seven GrTCS proteins docked with ABA (abscisic acid), were examined using the molecular docking technique and analyzed using the MOE tool.

| **Protein Name** | **S-score** | **RMSD-value** | **Bond distance**  **(Å)** | **Conserved residues** |
| --- | --- | --- | --- | --- |
| GrHK4.1 | -12.1191635 | 1.20003915 | 2.660 | MET 928 |
|  |  |  | 2.474 | ILE 925 |
|  |  |  | 3.294 | GLN 899 |
|  |  |  | 2.222 | LEU 900 |
|  |  |  | 2.517 | TRP 950 |
|  |  |  | 2.353 | HIS 902 |
|  |  |  | 2.925 | GLY 948 |
|  |  |  | 1.686 | THR 947 |
|  |  |  | 2.336 | GLU 929 |
|  |  |  | 2.084 | ALA 932 |
| GrHP4.2 | -9.83518314 | 1.57929456 | 1.604 | ARG 14 |
|  |  |  | 2.439 | GLU 31 |
|  |  |  | 2.130 | LEU 33 |
|  |  |  | 1.759 | GLN 34 |
|  |  |  | 2.793 | ASN 38 |
|  |  |  | 3.201 | THR 37 |
|  |  |  | 2.695 | PRO 39 |
| GrPHYA2 | -11.7826872 | 1.19056952 | 2.863 | ARG 411 |
|  |  |  | 2.860 | THR 412 |
|  |  |  | 2.149 | ASP 437 |
|  |  |  | 2.593 | VAL 439 |
|  |  |  | 2.489 | MET 436 |
|  |  |  | 2.150 | LYS 440 |
|  |  |  | 1.482 | LEU 959 |
|  |  |  | 2.136 | VAL 957 |
|  |  |  | 2.439 | LEU 438 |
|  |  |  | 2.007 | ASN 408 |
|  |  |  | 2.186 | GLY 955 |
|  |  |  | 2.666 | ASP 958 |
| GrPRR5.1 | -13.3339338 | 1.04909098 | 1.480 | GLN 475 |
|  |  |  | 2.220 | LEU 464 |
|  |  |  | 2.210 | LEU 478 |
|  |  |  | 2.356 | PHE 479 |
|  |  |  | 2.149 | PRO 482 |
|  |  |  | 1.878 | VAL 462 |
|  |  |  | 2.758 | THR 461 |
|  |  |  | 2.360 | MET 504 |
|  |  |  | 1.484 | THR 467 |
|  |  |  | 2.082 | GLN 466 |
| GrPRR5.2 | -11.4969902 | 1.07906568 | 2.951 | GLN 550 |
|  |  |  | 2.390 | THR 584 |
|  |  |  | 2.540 | VAL 565 |
|  |  |  | 1.454 | ARG 587 |
|  |  |  | 3.380 | SER 560 |
|  |  |  | 1.836 | ASN 547 |
|  |  |  | 1.260 | GLU 551 |
|  |  |  | 2.809 | ALA 542 |
|  |  |  | 2.128 | TYR 537 |
| GrPRR7.1 | -9.14681911 | 1.36022341 | 1.306 | SER 250 |
|  |  |  | 1.523 | SER 249 |
|  |  |  | 1.572 | ARG 321 |
|  |  |  | 2.702 | GLU 231 |
|  |  |  | 3.318 | THR 247 |
|  |  |  | 2.670 | SER 245 |
|  |  |  | 2.799 | ASN 232 |
| GrPRR9 | -6.74946165 | 2.34853554 | 2.189 | TYR 490 |
|  |  |  | 2.669 | GLY 491 |
|  |  |  | 2.698 | SER 527 |
|  |  |  | 1.713 | GLN 526 |
|  |  |  | 2.034 | GLN 503 |
|  |  |  | 2.254 | ASP 524 |
|  |  |  | 2.100 | LEU 532 |
|  |  |  | 2.374 | ALA 563 |
|  |  |  | 2.117 | HIS 539 |
|  |  |  | 2.063 | THR 559 |
|  |  |  | 1.502 | TYR 525 |
|  |  |  | 2.085 | GLN 506 |
